# Optogenetic stimulation of the median preoptic nucleus: Effects on hypothalamic paraventricular nucleus magnocellular neurons after chronic intermittent hypoxia exposure

**DOI:** 10.1111/jne.70100

**Published:** 2025-10-19

**Authors:** Obed T. Paundralingga, Shuping Jia, George E. Farmer, Glenn M. Toney, J. Thomas Cunningham

**Affiliations:** ^1^ Department of Physiology and Anatomy University of North Texas Health Fort Worth Texas USA; ^2^ Department of Anatomy, Faculty of Medicine Universitas Brawijaya Malang Jawa Timur Indonesia; ^3^ Department of Medical Physiology Texas A&M University College of Medicine Bryan Texas USA

**Keywords:** chronic intermittent hypoxia, hypothalamus, optogenetics, plasticity, preoptic area

## Abstract

Median preoptic nucleus (MnPO) neurons projecting to the hypothalamic paraventricular nucleus (PVN) are linked to hypertension induced by chronic intermittent hypoxia (CIH), a model of obstructive sleep apnea. The modulation of MnPO‐driven synaptic activity in PVN magnocellular neurons (MNCs) by CIH remains unexamined. We hypothesized that single and repetitive activation of MnPO–PVN projections causes differential synaptic plasticity in MnPO–PVN synapses with and without CIH exposure. Adult male rats were prepared using an intersectional viral approach to induce Cre‐dependent channelrhodopsin expression in PVN‐projecting MnPO neurons. Two weeks after stereotaxic surgery, some rats were exposed to 7 days of CIH. All rats were anesthetized and their brains were prepared for in vitro electrophysiological recording from PVN MNCs and optogenetic stimulation of the MnPO. We observed distinct EPSC and IPSC response patterns to the optogenetic stimulation of the MnPO. Low‐frequency optogenetic stimulation (15 Hz) resulted in short‐term potentiation manifested in increased poststimulatory spontaneous EPSC (sEPSC) frequency without altering amplitude while gradually increasing poststimulatory sIPSC frequency and amplitude, shifting some neurons to a more inhibitory state. CIH increased the amplitude of both sEPSCs and stimulation‐evoked EPSCs while reducing their frequency. In contrast, CIH enhanced both the amplitude and frequency of sIPSCs and stimulation‐evoked IPSC. Stimulation‐evoked currents recorded during train protocols reflected a mixture of spontaneous and evoked events. Optogenetic stimulation increased the intrinsic excitability of MNCs in rats exposed to CIH. Activation of the MnPO–PVN pathway recruits both excitatory and inhibitory synaptic circuits converging onto PVN MNCs. CIH induces metaplasticity within this pathway, manifested as strengthened excitatory synaptic drive and heightened intrinsic excitability of PVN MNCs, which is counterbalanced by an adaptive increase in inhibitory tone. These parallel changes could explain why CIH is not associated with increased neurohypophysial hormone release.

## 1 INTRODUCTION

The median preoptic nucleus (MnPO) is located dorsal, rostral, and ventral to the midline anterior commissure in the anteroventral third ventricle (AV3V) region of the forebrain.^1^, ^2^, ^3^, ^4^ The MnPO contributes to a diversity of vital functions, including thermoregulation, sleep, body fluid balance, and cardiovascular regulation.^1^, ^2^, ^3^, ^4^, ^5^, ^6^ Neurons from two circumventricular organs (CVO) of the lamina terminalis—the subfornical organ (SFO) and organum vasculosum laminae terminalis (OVLT)—supply glutamatergic input to the MnPO.^7^, ^8^ SFO and OVLT neurons detect changes in plasma osmolality and circulating hormones, such as angiotensin II (Ang II).^9^, ^10^, ^11^ Direct neural projections to the MnPO from the brainstem nucleus of the solitary tract (NTS) and A1 noradrenergic cell group relay signals to the MnPO related to body fluid balance and blood pressure.^2^, ^6^ MnPO sends glutamatergic and GABAergic projections to the supraoptic nucleus (SON) magnocellular neurosecretory cells (MNCs) and paraventricular nucleus (PVN)^12^, ^13^, ^14^, ^15^, ^16^, ^17^ parvocellular neurons and MNCs. These projections allow the MnPO to influence the pituitary release of arginine vasopressin (AVP) and oxytocin (OT) to influence cardiovascular and neuroendocrine functions.^17^, ^18^, ^19^, ^20^, ^21^ In addition to the above largely acute functions, the MnPO participates in different models of hypertension.^22^, ^23^, ^24^, ^25^

One of these hypertension models, chronic intermittent hypoxia (CIH), mimics the hypoxemia associated with obstructive sleep apnea (OSA).^26^, ^27^, ^28^ In OSA, repeated total or partial airway obstruction leads to interruptions in breathing during sleep, resulting in episodes of arterial hypoxemia.^29^ Notably, more than half of patients with OSA experience daytime hypertension and attenuated reductions in blood pressure during sleep.^30^, ^31^, ^32^, ^33^ In CIH, exposure to bouts of intermittent hypoxia contributes to systemic hypertension in male rats that is maintained throughout the normoxic awake period,^28^, ^34^, ^35^ comparable to diurnal hypertension found primarily in male patients with OSA.^36^, ^37^ Our lab recently reported that the development of a sustained increase in blood pressure during normoxia depends on the integrity of PVN‐projecting MnPO neurons, while the blood pressure increase during the hypoxic phase is less dependent on it.^38^, ^39^ Notably, not only does CIH increase the expression and activation of MnPO Ang II type 1 receptors (AT1aR) and the expression of FosB,^40^, ^41^ sustained CIH hypertension is abrogated by interrupting MnPO AT1aR function.^40^, ^42^

The PVN contains several distinct functional populations of neurons that could contribute to chronic intermittent hypoxia (CIH)–induced hypertension. Based on electrophysiological characteristics, PVN neurons can be categorized as Type I (magnocellular/MNCs), Type II (parvocellular), and Type III (pre‐autonomic parvocellular).^43^, ^44^ In mice, 15 Hz optogenetic stimulation of the MnPO–PVN pathway has been shown to increase blood pressure, in part, due to the activation of MNCs and their associated vasopressin release.^20^ Although the sustained increase in blood pressure observed in our CIH model does not appear to be vasopressin‐dependent,^45^ it is possible that interactions between MNCs and preautonomic neurons support this sustained increase.^46^

The expression levels of c‐Fos and FosB are linked to changes in synaptic plasticity and neural excitability,^47^, ^48^, ^49^, ^50^, ^51^ with c‐Fos generally associated with acute increases in synaptic activity and FosB with more complex synaptic effects associated with chronic or intermittent stimulation.^25^, ^52^, ^53^ c‐Fos and FosB have been detected in parvocellular and magnocellular neurons following CIH exposure.^45^, ^54^ A study also demonstrated that the number of c‐Fos positive cells increased in the central magnocellular region of the PVN in response to 10 repetitive bouts of hypoxia in acute intermittent hypoxia (AIH), particularly when hypoxic episodes were more severe (6% O_2_),^55^ while five bouts were equally effective in inducing post‐exposure sympathetic plasticity.^56^ There is limited information on how CIH and activation of MnPO inputs affect the synaptic plasticity of PVN magnocellular neurons.

Based on the contribution of PVN‐projecting MnPO neurons to CIH‐induced hypertension in male rats, we hypothesized that single or repetitive trains of MnPO–PVN pathway‐specific optogenetic activation would enhance synaptic strength, frequency, and excitability in PVN MNCs, with prior CIH exposure modulating this response. An intersectional adeno‐associated viral (AAV) optogenetic approach was used to selectively express channelrhodopsin in MnPO neurons projecting to the PVN. Whole‐cell patch‐clamp recordings were obtained from PVN neurons characterized by their electrophysiological properties.^43^, ^44^ The cells were tested for their responses to repeated optogenetic stimulation of the MnPO using a protocol based on previous stimulation and AIH studies^20^, ^57^ and the in vivo activity of MnPO neurons.^18^ In our preliminary studies, we initially observed that optogenetic stimulation of MnPO affected both MNCs and non‐MNC PVN neurons. Based on the complexity of our results, the current study focuses on the effects of optogenetic stimulation of PVN afferents from MnPO on PVN magnocellular neurons.

## 2 MATERIALS AND METHODS

### 2.1 Animal and housing

Experiments used a total of 68 adult (250–300 g) male Sprague–Dawley rats (Charles River Laboratory, Wilmington, MA). Animals were individually housed in temperature‐controlled rooms with a 12‐h light–dark cycle, with the light phase lasting from 7:00 AM to 7:00 PM. Plastic housing cages were connected to a closed‐air filtration system. Cages contained corn cob bedding and shredded paper for enrichment. Rats had ad libitum access to water and standard rat chow (LabDiet, St. Louis, MO). Rats were given 1 week to acclimate before experiments began. Experiments were performed according to the National Institutes of Health Guide for the Care and Use of Laboratory Animals (8th edition) using protocols approved by the University of North Texas Health Science Center Institutional Animal Care and Use Committee.

### 2.2 Viral constructs

To induce a Cre‐dependent expression of channelrhodopsin (ChR2) in PVN‐projecting MnPO neurons, this study used AAV1‐EF1a‐double floxed‐hChR2(H134R)‐mCherry‐WPRE‐HGHpA (Addgene #20297‐AAV1, titer 7 × 10^12^ vg/mL), and AAV9‐hSyn‐HI.eGFP‐cre‐WPRE‐SV40 (Addgene #105540‐AAV9, titer 1 × 10^13^ vg/mL). While the AAV9 Cre vector injected into the PVN (200 nL/site) has anterograde and retrograde capacities, the Cre‐dependent ChR2 vector injected into the MnPO (100 nL/site) specifically ensured ChR2 expression only in the MnPO–PVN pathway. In some experiments, a retrogradely transported AAV expressing tdTomato, AAVrg‐CAG‐tdTomato (Addgene #59462‐AAVrg, titer 7 × 10^12^ vg/mL), was targeted to the rostral ventrolateral medulla (RVLM) to aid visual differentiation of Type I and Type III (preautonomic) PVN neurons (50 nL/site). Viruses were used undiluted. All viral constructs included a fluorescent protein reporter to allow verification of injection sites.

### 2.3 Stereotaxic surgery

**FIGURE 1:**
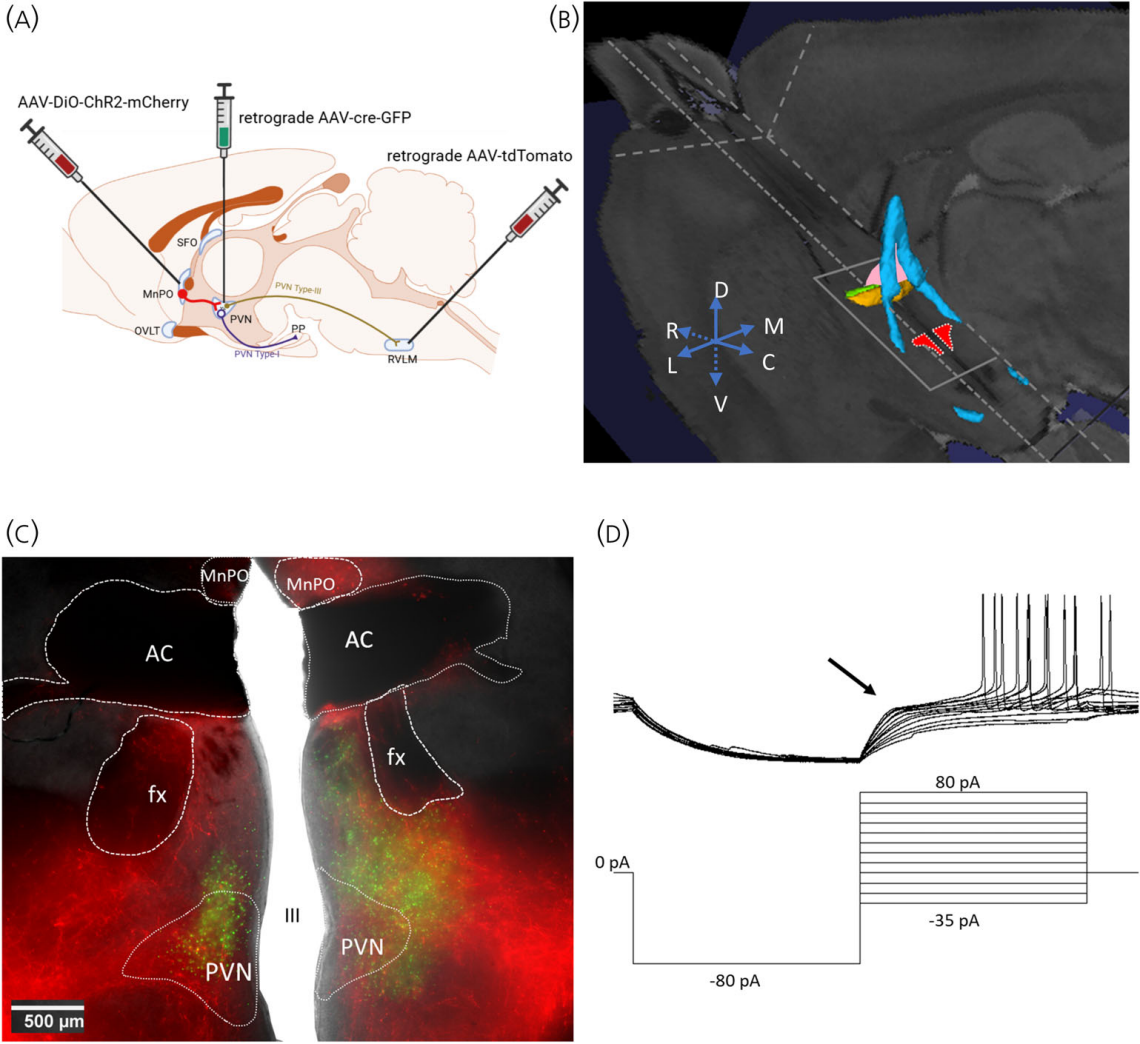
(A) Schematic diagram illustrating the Virus injections. The PVN was injected with retrograde Cre virus AAV9.hSyn.HI.eGFP‐cre.WPRE.SV40 bilaterally while the MnPO was injected with Cre‐dependent Channelrhodopsin virus AAV1‐EF1a‐double floxed‐hChR2(H134R)‐mCherry‐WPRE‐HGHpA. In some experiments, a retrogradely transported AAV expressing tdTomato, AAVrg‐CAG‐tdTomato, was targeted to the rostral ventrolateral medulla (RVLM) to aid visual differentiation of Type I and Type III (preautonomic) PVN neurons. AC, anterior commissure; fx, fornix; III, 3rd Ventricle; PVN, paraventricular nucleus of the hypothalamus; MnPO, median preoptic nucleus. (B) A digital 3D rendering illustrating the oblique plane of section for brain slices based on the Waxholm Space Atlas of the Sprague Dawley Rat Brain^107^ (RRID:SCR_017124). The relevant structures are delineated in color: PVN (red), MnPO (pink), fx (blue), AC (green and yellow). Note that the plane is angled 33° from the horizontal flat surface of the skull. (C) Digital images of bisected oblique brain slices containing the MnPO and the PVN that were used for electrophysiology experiments. This section was taken from approximately the position depicted by the gray rectangle in part figure B. (D) Example of the identification of PVN MNCs. Representative membrane potential traces showing a Type I PVN neuron with delay to spike onset caused by the dampening of the depolarization using current clamp recordings (arrow) in response to a series of depolarizing injected currents delivered at a hyperpolarized membrane potential.

Stereotaxic injections were made using calibrated borosilicate glass capillary tubes (Drummond Scientific Company, Broomall) pulled to a final inner tip diameter of 1 μm using a horizontal puller (Model P‐2000, Sutter Instrument Co, USA). The injector was connected to a 5 mL syringe via an aspirator tube. Before surgery, rats were anesthetized with isoflurane (2%–3% in O_2_), and their scalps and nuchal regions were shaved and disinfected with alcohol and iodine. Each rat was placed in a stereotaxic head frame equipped to maintain isoflurane anesthesia (David Kopf, Tujunga, CA). A midline scalp incision was made to expose the skull. To ensure accurate injections, skulls were leveled between cranial suture landmarks bregma and lambda.^58^ After burr holes were drilled, a glass injector pipette was lowered into PVN at the following coordinates: 1.8 mm caudal and ±0.25 mm lateral to bregma, 7.6 mm ventral from dura (Figure 1A).^58^ Retrograde AAV‐Cre (200 nL) was then bilaterally injected into PVN at a rate of 200 nL/min. The injection volume was determined visually from calibrated markings on the pipette. Cre‐dependent ChR2 viral (100 nL) was injected into MnPO at the following coordinates using an angled approach (8° from vertical): 0.25 mm caudal and 0.9 mm lateral from bregma, 6.7 mm ventral from the surface of the skull (Figure 1A).^39^ Retrograde AAV encoding tdTomato was injected into the RVLM by laterally reflecting the nuchal muscles, opening the atlantooccipital membrane. An injection pipette was lowered to the RVLM using the following coordinates: 1.8 mm rostral, 1.8 mm lateral, and 2.9 mm ventral from calamus scriptorius with an angled approach (20° rostral, Figure 1A).^59^ AAV was delivered in a volume of 50 nL. After each injection, pipettes were left in place for 5 min to allow for diffusion of viral particles before being slowly withdrawn. Gel foam was packed into each burr hole, and absorbable antibiotic sutures were used to close the incision site to minimize postsurgical infection. A nonsteroidal anti‐inflammatory drug, carprofen (Rimadyl, 2 mg orally), was given before and after surgery for pain management. All rats were given at least 3 weeks for surgical recovery and to allow for robust expression of viral genes before acute experiments were performed.

### 2.4 Chronic intermittent hypoxia

Two weeks after stereotaxic surgery, some rats were transferred to CIH chambers. Rats were individually housed in a 20 cm × 23 cm cage placed inside plexiglass chambers. The CIH protocol, generated using OxyCycler A84XOV Dynamic O_2_ Animal Chamber Controller (BioSpherix, Ltd., RRID: SCR_021185), consisted of 6‐min cycles: 3 min of 21% oxygen room air pumped in, 90 s of nitrogen pumped in to lower the chamber O_2_ to 10% oxygen, and then 90 s of maintenance at 10% oxygen. This cycle, repeated 10 times per hour for 8 h a day (8:00 AM to 4:00 PM) for 7 consecutive days, has been shown to induce the development of mild hypertension in rats.^25^, ^39^ The morning after the CIH exposure protocol ended, rats were euthanized for electrophysiology studies. Normoxic controls were housed in the same rooms but exposed only to room air while housed in standard cages.

### 2.5 Brain slice preparations

Three weeks following stereotaxic surgery, which for some animals included 7 days of CIH exposure, rats were anesthetized with isoflurane (2%), decapitated, and their brains were prepared for patch‐clamp recording as previously described.^41^ Brains were extracted and submerged in ice‐cold carbogenated (95% O_2_, 5% CO_2_) solution consisting of (in mM): 3.0 KCl, 1.0 MgCl_2_ · 6H_2_O, 2.0 CaCl_2_, 2.0 MgSO_4_, 1.25 NaH_2_PO_4_, 26 NaHCO_3_, 10 d‐glucose, and 206 sucrose (300 mOsm/L, pH 7.4). Oblique slices (300 μm, angled 33° from the level plane of the ventral aspect of the brain) containing the MnPO and the PVN were cut using a Microslicer DTK Zero 1 (Ted Pella, Inc., Redding, CA) and bisected at the midline (Figure 1B,C). Next, slices were incubated at room temperature in oxygenated artificial cerebrospinal fluid (aCSF) containing (in mM): 126 NaCl, 3.0 KCl, 2.0 CaCl_2_, 2.0 MgSO_4_, 1.25 NaH_2_PO_4_, 26 NaHCO_3_, 10 D‐glucose (300 mOsm, pH 7.4) for a minimum of 1 h before recording.

### 2.6 Electrophysiology

Brain slices containing the MnPO and PVN were transferred to a submersion recording chamber and superfused with 5% CO_2_–95% O_2_ equilibrated aCSF (room temperature, 2–2.5 mL/min). Slices were visualized using an upright microscope equipped with epifluorescence and differential interference contrast optics (BX50WI, Olympus). Appropriate filters were used to visualize fluorescently labeled neuronal soma. A horizontal micropipette puller (P‐1000 Flaming Brown, Sutter Instruments, Novato, CA) was used to fabricate recording pipettes (4–6 mΩ) from borosilicate glass capillaries (Kwik‐Fil TM, World Precision Instrument, Sarasota). Recording pipettes were filled with a solution containing (in mM): 145 K‐gluconate, 1 MgCl_2_ · 6H_2_O, 10 HEPES, 1.1 EGTA, 2 Mg_2_‐ATP, 0.3 Na_2_‐GTP (osmolarity 280–285 mOsmol/L; pH 7.3).

Cells were characterized as Type I, Type II, or Type III PVN neurons based on the delay to spike onset caused by the dampening of the depolarization using current clamp recordings (Figure 1D).^43^ Type I PVN neurons were located in subnuclei of the PVN that contain magnocellular neurosecretory cells, had pronounced transient *V*_*m*_ depolarization, and lacked low threshold spikes. These neurons were patched regardless of their physical apposition to the mCherry/EGFP‐labelled MnPO terminals in the PVN. Whole‐cell voltage‐ and current‐clamp recordings were amplified and digitized using a MultiClamp 700B and a Digidata 1440A, respectively (Molecular Devices, San Jose, CA). Signals were filtered at 2 kHz, digitized at 10 kHz, and saved on a computer for offline analysis. After achieving a giga‐ohm seal and the whole‐cell configuration, cell capacitance, access resistance, and resting membrane potential (*V*_*m*_) were monitored until stable.

Recordings were collected over a total period of 40 min, consisting of alternating baseline recordings and optogenetic stimulation trains. Each stimulation train consisted of continuous 15 Hz LED‐generated blue light pulses (473 nm, pulse width 20 ms, duty cycle 30%) delivered for 1 min.^20^ Within this 1‐min stimulation, there were 0.37 s stimulation off‐times between 10 s sweeps. The stimulation train was repeated five times, with intervening 5‐min periods.^60^ Five‐minute baseline recordings were collected before the first and after the last stimulation train. Before the first stimulation train, between consecutive trains, and after the last train, MnPO afferents were photostimulated at a lower frequency (0.066 Hz, with one pulse of 5 ms duration every 15 s) to monitor evoked postsynaptic currents. Optogenetic stimulation trains were delivered via a fiber‐optic cannula placed over the PVN area of the hemi‐slice to activate MnPO axons in the PVN (Figure 1C).

Excitatory and inhibitory postsynaptic currents (EPSCs and IPSCs) were simultaneously recorded in voltage‐clamp mode. The *V*_hold_ was set at −70 mV. The liquid junction potential was calculated offline to be −15.78 mV,^61^ putting the actual *V*_hold_ at approximately −54 mV. The amplitude of each current was measured as the difference between its event baseline (averaged over a 10‐ms search window) and its peak, while the interevent interval was defined as the time between successive peaks. Effects of intermittent optogenetic stimulation on the frequency (represented by IEI) and amplitude of synaptic currents were determined by comparing events recorded before, during, and after the stimulation train. Events were detected by setting a current amplitude threshold above the noise level defined as 3× the standard deviation of the Im signal recorded in the absence of evoked synaptic responses. The sodium channel blocker tetrodotoxin was not included in our bath solution to allow optogenetic stimulation of the MnPO to activate the PVN. In preliminary studies, the EPSCs and IPSCs were pharmacologically characterized using CNQX (10 μM, Tocris, Minneapolis, MN) and Gabazine (25 μM, Tocris, Minneapolis, MN).

Evoked EPSCs and IPSCs were defined as the downward (EPSC) or upward (IPSC) deflections in postsynaptic current recorded in response to optogenetic stimulation(s). The latency of baseline stimulation was defined as the interval between the onset of the stimulation pulse and the onset of the evoked postsynaptic current (eEPSCs and eIPSCs) during the periods outside of the stimulation trains (i.e., baseline/interstimulus periods). In some neurons, the postsynaptic current latency was also measured during the stimulation train period, using the onset of the first pulse of the stimulation train as the initial landmark. Jitter was defined as the standard deviation of latency.^62^ During the stimulation trains, the currents were not always time‐locked to the optogenetic pulses. This variability could have been due to the specific localization of channelrhodopsin in presynaptic neurons in MnPO and the complexity of the circuit architecture. For some statistical analyses, we examined the PSCs recorded before and after stimulation. When we compared the PSCs that occurred during the stimulation trains (“stimulation‐evoked” currents), all of the currents that occurred during the stimulation period, including spontaneous PSCs.

The effect of CIH on the intrinsic excitability of MNCs was tested in current‐clamp mode before the first baseline recording and 5 min after the fifth stimulation train. Currents were injected through the patch electrode in 10 pA increments from −25 pA to +65 pA, each for a duration of 200 ms to achieve steady‐state firing, including any development of spike frequency adaptation, following a baseline of no current injection. Current‐clamp recordings were performed without drugs added to the aCSF.

### 2.7 Data analysis

EPSCs and IPSCs were analyzed offline using Easy Electrophysiology v2.6.1 (Easy Electrophysiology Ltd., London, UK). Statistical comparisons were made using IBM SPSS version 30.0 (IBM Corp, Armonk, NY) and visualized using OriginPro, Version 2025 (OriginLab Corporation, Northampton, MA). Each identified Type I neuron/MNC was classified as either responsive or non‐responsive to optogenetic stimulation of the MnPO. Responsive neurons were further analyzed according to different response patterns based on the combination of excitatory and inhibitory currents evoked by blue‐light pulses during baseline stimulation and repeated stimulation trains. Excitatory and inhibitory currents were analyzed separately.

To examine the effects of CIH, optogenetic stimulation trains, and optogenetic response groups, we conducted a Generalized Linear Mixed Model (GLMM) analysis of the impacts of hypoxic status (normoxic/NORM and CIH‐exposed/CIH), optogenetic stimulation (Prestimulation/Prestim‐, Poststimulation/Poststim‐), and recorded response patterns (IPSC‐EPSC sequence, EPSC‐IPSC sequence, EPSC only, IPSC only).^63^ The interaction between hypoxic status and optogenetic stimulation, and between optogenetic stimulation and response pattern, was analyzed as fixed effects. Neurons nested within the fixed effect groups served as random effects to account for within‐subject variability. The dependent variables were EPSC or IPSC absolute amplitude (i.e., amplitude value converted to its absolute value to fit gamma distribution for GLMM procedures), EPSC or IPSC inter‐event interval/IEI, EPSC or IPSC kinetic parameters, and IPSC/EPSC charge transfer ratio obtained from events recorded 1 min before the first and after the last stimulation train (spontaneous currents: Baseline/Prestim‐01, Poststim‐05). The model used a gamma distribution with a log link function for the continuous outcome variable and included a random slope for neurons within groups, with a variance components covariance structure for the random effects. To further explore the differences between groups, we conducted pairwise comparisons of the estimated marginal means (EMMs) using Bonferroni correction to adjust for multiple comparisons. The Bonferroni correction was applied to control the family‐wise error rate. A *p* value of < .05 was required for significance. Two‐way ANOVA was used to assess changes in excitability within NORM and CIH groups, with stimulation condition (Prestim‐01 vs. Poststim‐05) as the between‐group factor and current injection as the repeated measure; the same approach was applied for input resistance analysis. All data are expressed as the mean ± standard error (SE). The analyses of dependent variables for EPSC or IPSC absolute amplitude and IEI from before and after intermediate stimulation trains^2^, ^3^, ^4^ were reported in the supplement.

## 3 RESULTS

### 3.1 MnPO optogenetic activation produces multiple patterns PVN neuronal response

**FIGURE 2:**
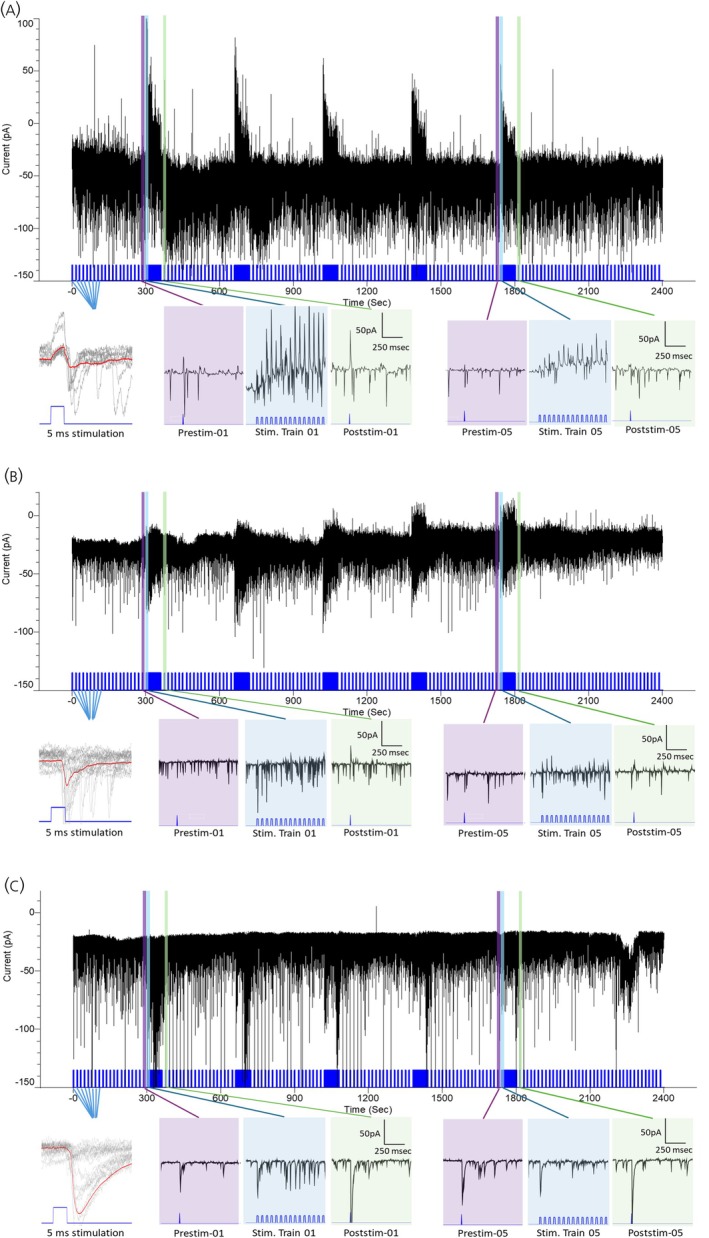
Representative individual examples demonstrating response patterns to optogenetic stimulation of the MnPO–PVN pathway on electrophysiologically characterized PVN MNCs. (A–C) Upper panel: 40‐min raw voltage‐clamp recording from a representative neuron demonstrating the effects of repeated 15 Hz optogenetic stimulation trains on postsynaptic currents. Before the first stimulation train (473 nm, pulse width 20 ms, duty cycle 30%), between consecutive trains, and after the last train, the neuron was stimulated once every 15 s with a 5 ms light pulse to monitor the evoked currents. Lower panels: (left) Evoked response to the 5 ms optogenetic stimulations; (middle and right) 1‐s expanded segments from the upper panel before, during, and after the first stimulation train and the fifth stimulation train with their respective timescales (right). Blue marks in the upper and lower panels indicate the timing of optogenetic stimulations of the MnPO. (A) IPSC/EPSC sequence response. Single optogenetic stimulation pulses and repeated stimulation trains evoked IPSCs followed by EPSCs. (B) EPSC/IPSC sequence response. Single optogenetic stimulation pulses evoked EPSCs. Initially, optogenetic stimulation trains elicited EPSCs; however, as the stimulation train progressed, IPSCs gradually emerged. (C) EPSC‐only response. Single optogenetic stimulation pulses and repeated stimulation trains evoked only EPSCs.

In PVN MNCs, blue light stimulation evoked postsynaptic currents both from cells from normoxic (*n* = 14 cells, 9 animals) and CIH‐exposed rats (*n* = 11 cells, 7 animals) (Figure 2A–C), with different response patterns observed across groups. In one group of cells (*n* = 6 cells, 5 animals), 5 ms single pulse stimulation and the 15 Hz stimulation trains evoked both IPSCs and EPSCs sequentially (IPSC/EPSC sequence: EPSC latency = 8.03 ± 0.73 ms, EPSC jitter = 2.76 ± 0.89 ms, IPSC latency = 4.99 ± 1.28 ms, IPSC jitter = 0.905 ± 0.252 ms, Figure 2A). In the second group (*n* = 7 cells, 5 animals), only EPSCs were observed in responses to single 5 ms pulses, but EPSCs and IPSCs were observed during subsequent 15 Hz MnPO efferent stimulation trains (EPSC/IPSC sequence: EPSC latency = 5.53 ± 0.49 ms, EPSC jitter = 1.576 ± 0.439 ms, IPSC latency = 136.36 ± 62.15 ms, IPSC jitter = 132.9 ± 71.13 ms, Figure 2B). A third group of cells (*n* = 10 cells, 9 animals) exhibited only EPSCs in response to single pulse and repeated 15 Hz stimulation trains (EPSC only: EPSC latency = 6.8 ± 0.5 ms, EPSC jitter = 1.526 ± 0.459 ms, Figure 2C). We observed only one neuron that exhibited only evoked IPSCs throughout the entire protocol. Another neuron shifted from exhibiting only EPSCs before the first stimulus train to only IPSCs after the train. Average EPSC latencies were significantly different among the groups, most notably between response sequences IPSC/EPSC and EPSC/IPSC (*F*(2, 1588) = 3.67, *p* = .22, GLMM).

Bath application of the AMPA receptor antagonist CNQX abolished EPSCs (51.7 ± 11.2 pA to 0.0 pA; *t* ^4^ = 4.63, *p* = .009; Figure S1). Likewise, the GABA_A_ receptor antagonist Gabazine eliminated IPSCs (13.8 ± 1.16 pA to 0.0 pA; *t* ^4^ = 11.9, *p* = .0003; Figure S1). These results indicate that optogenetically evoked responses were primarily glutamatergic and GABAergic.

**FIGURE 3:**
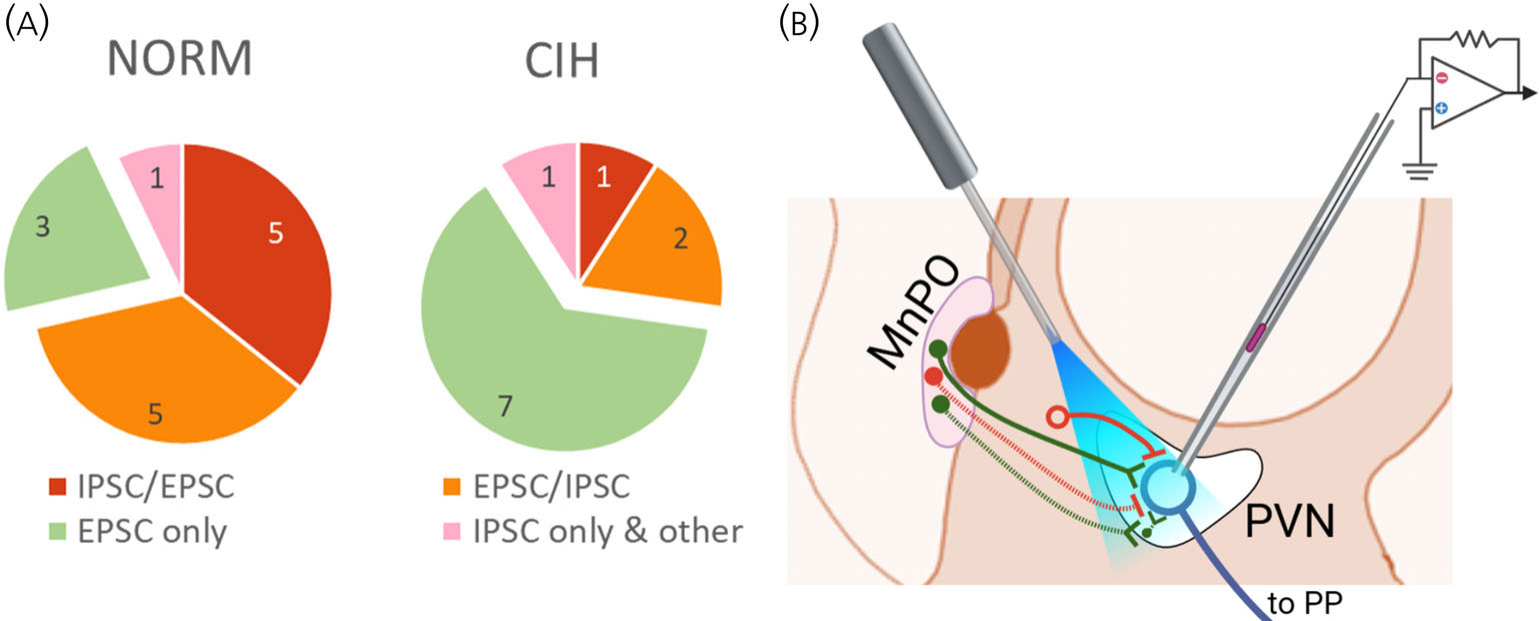
Response pattern proportion and analysis of IPSC difference. (A) The proportion of different response pattern groups under normoxia and CIH. The exploded slice in green is the response pattern with EPSC only. The number indicates the number of recorded neurons for each group. Fisher exact test, *p* = .0486. (B) Summary diagram illustrating different hypothetical MnPO to PVN projections that could account for the different response patterns of PVN MNCs. One mechanism for the observed IPSC/EPSC sequences could result from the simultaneous activation of both monosynaptic inhibitory (dotted red neuron) and polysynaptic excitatory projections to the MnPO projection (dotted green neurons). This could account for an initial inhibitory response, followed by a delayed excitation. This is supported by the differences in the latencies of the IPSCs and EPSCs. The EPSC/IPSC sequence may result from the activation of excitatory MnPO neurons (solid green neuron) and the subsequent recruitment of Non‐MnPO GABAergic neurons (red neuron with empty soma) through nonsynaptic mechanisms such as NO or peptide release. Cells that responded with only EPSC could be responding to direct excitatory projections from the MnPO (solid green neuron), while cells that responded with only IPSCs could be responding to direct inhibitory projections from the MnPO (dotted red neuron). MnPO, median preoptic nucleus; PP, posterior pituitary; PVN, paraventricular nucleus of the hypothalamus.

There was a change in the distribution of these response patterns associated with CIH. There was a significant increase in the number of cells that displayed only EPSC responses, as compared with the normoxic control distribution (Fisher exact test, NORM: IPSC *n* = 11 cells, EPSC‐only *n* = 3 cells; CIH: with IPSC *n* = 4 cells, EPSC only *n* = 7 cells; *p* = .0486; Figure 3A). Due to this shift in the number of cells with specific response patterns, proper statistical comparisons of the difference between normoxic and CIH conditions for each response pattern group could not be performed. The neuron receiving only IPSCs during stimulation was also excluded from the statistical analysis.

Under normoxic conditions, there were no significant differences in the amplitude or frequency of sEPSCs and stimulation‐evoked EPSCs among all groups with EPSC response (Figure S2A–D). Although the amplitudes of IPSCs were similar between IPSC/EPSC and EPSC/IPSC sequence groups (Figure S3A,C), the frequency of IPSCs displayed several notable differences. The frequency of IPSCs before the first stimulation trains was consistently higher in the IPSC/EPSC sequence group (Figure S3B). This pattern was also evident in the frequency of sIPSCs during the first stimulation train (Figure S3D). However, after multiple stimulation trains, the frequency of sIPSCs in the EPSC/IPSC sequence group increased to a level comparable to that of the IPSC/EPSC sequence group (Figure S3B). The observed differences in latency and jitter between the IPSC/EPSC and EPSC/IPSC sequence groups, along with the varying effectiveness of optogenetic stimulation in evoking IPSCs and the progressive recruitment of sIPSCs between stimulation trains, suggest potential underlying differences in the synaptic sources and modulatory mechanisms associated with the responses to optogenetic stimulation to the PVN MNCs (Figure 3B).

### 3.2 Effects of optogenetic stimulation on EPSC, IPSC, and IPSC/EPSC ratio

#### 3.2.1 Effects of optogenetic stimulation on spontaneous EPSC amplitude and frequency

**FIGURE 4:**
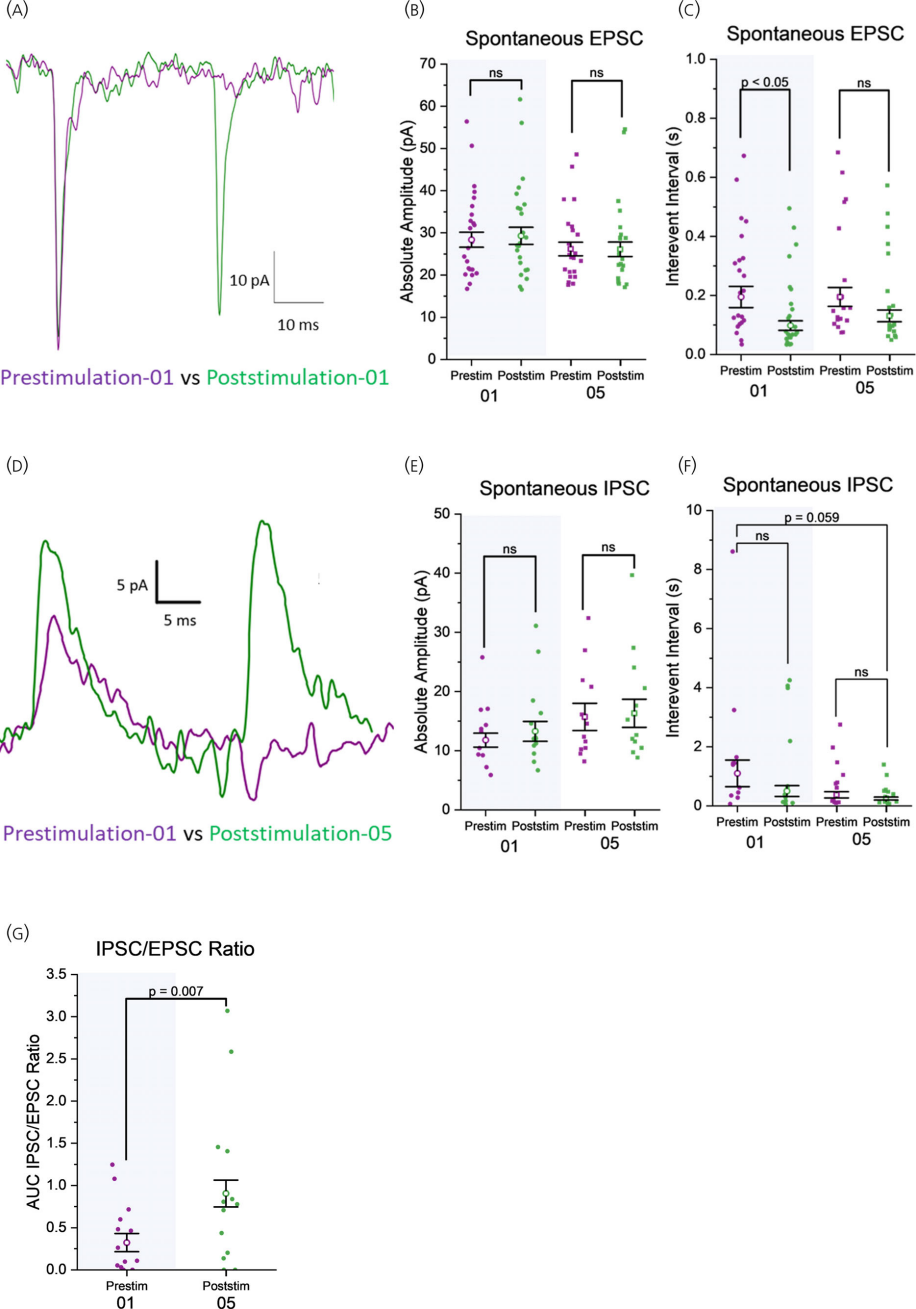
Optogenetic stimulation of the MnPO–PVN pathway increases the frequency of spontaneous EPSCs (sEPSCs) in PVN MNCs but does not affect their amplitude. Following repeated stimulation trains, PVN MNCs exhibit a trend toward increased spontaneous IPSC (sIPSC) frequency and amplitude. sEPSC and sIPSC frequency are denoted by their interevent interval (IEI). (A) Representative sEPSCs recorded from a PVN MNC demonstrating the effects of optogenetic stimulation train. (B) No significant difference was observed in the amplitude of sEPSCs after the first (*F*(1, 27,858) = 0.118, *p* = .732) or fifth (*F*(1, 19,604) = 0.001, *p* = .975) optogenetic stimulation trains. (C) Optogenetic stimulation acutely decreased sEPSC IEI significantly after the first stimulation train (*F*(1, 27,848) = 9.181, *p* = .007). The effect of optogenetic stimulation waned by the fifth stimulation train (*F*(1, 19,600) = 3.537, *p* = .071). EPSC amplitude and IEI, *n* = 23 cells, 14 animals. (D) Representative sIPSCs recorded from PVN MNC demonstrating the effects of optogenetic stimulation train. (E) sIPSC amplitudes remained unchanged after the first (*F*(1, 5258) = 0.54, *p* = .621) and fifth stimulation trains (*F*(1, 6326) = 0.034, *p* = .853). (F) IEI remained unchanged after the first (*F*(1, 5253) = 2.072, *p* = .217) and fifth stimulation train (*F*(1, 6323) = 1.449, *p* = .273) but showed a decreasing tendency by the end of the fifth compared with prestimulation (*F*(1, 5399) = 10.692, *p* < 0.001 GLMM, *p* = .059 pairwise comparison). IPSC amplitude and IEI: *N* = 15 cells, 11 animals. (G) Repeated stimulation trains led to a significant increase in spontaneous IPSC/EPSC ratio (*F*(1, 20) = 7.421, *p* = .007) (i.e., ratio of the cumulative IPSC area‐under‐curve to the cumulative EPSC area‐under‐curve). *n* = 15 cells, 11 animals. IEI = inter‐event interval (i.e., 1/frequency). Prestim‐01/05 = 1‐min prestimulation baseline 01/05. Poststim‐01/05 = 1‐min poststimulation 01/05. GLMM. Data are presented as estimated marginal mean (empty symbols) ± SE, with individual data plots (filled symbols) representing the mean for each neuron.

To evaluate the effect of single and repeated 15 Hz optogenetic stimulation trains of the MnPO–PVN pathway on sEPSC amplitude and frequency, we compared these EPSC parameters before and after each stimulation train across all response pattern groups. Since optogenetic stimulation produced comparable effects in both normoxic and CIH neurons, we combined the data from these two conditions for reporting (*n* = 23 cells, 14 animals). Our findings indicate that optogenetic stimulation trains did not acutely alter the amplitude of sEPSCs (Figures 2, 4A,B, and S4A). However, the first stimulation trains increased the frequency of sEPSCs (i.e., decreased sEPSC IEI. Prestim‐01: 0.223 ± 0.036 s, Poststim‐01: 0.116 ± 0.017 s; Figures 2 and 4A,C). Although there was a trend for increasing sEPSC frequency after the fifth stimulation train, the difference was not significant (Figures 4C and S4B). In conclusion, while optogenetic stimulation trains do not affect the amplitude of sEPSCs, they increase poststimulatory sEPSC frequency, though their efficacy diminishes with repeated stimulations.

#### 3.2.2 Effects of optogenetic stimulation on spontaneous IPSC amplitude, frequency, and IPSC/EPSC ratio

To determine whether optogenetic stimulation of MnPO terminals had pre‐ or postsynaptic mechanisms/sites of action on PVN magnocellular neuron IPSCs, we compared the amplitude and frequency of sIPSCs before and after each stimulation train across all neurons with evoked IPSC responses in normoxic and CIH neurons (*n* = 15 cells, 11 animals). We found that optogenetic stimulation did not acutely increase poststimulatory sIPSC amplitude (Figures 4E and S5A), similar to the lack of an immediate effect of the optogenetic stimulation train on sEPSC amplitude. In contrast to the effect of stimulation on sEPSC frequency, where a notable increase was observed after the first stimulation train, the change in sIPSC frequency was not significant after each stimulation train (Figures 4F and S5B). Although stimulation trains did not produce immediate poststimulatory effects, the frequency of sIPSCs exhibited a progressive increase beginning after the second train and continuing through the last stimulation train, relative to baseline values recorded prior to the first stimulation (Figures 4D,F and S5B).

To explore further if repeated optogenetic stimulation to PVN afferents from MnPO affected the excitatory and inhibitory balance of PVN MNCs that displayed both eIPSCs and eEPSCs (*n* = 15 cells, 11 animals), we calculated the sIPSC/sEPSC ratio for each neuron. The ratio between IPSC and EPSC amplitude was calculated as the ratio of cumulative charge transfer of sIPSC to that of the sEPSC recorded over the same time interval. The ratio was obtained from two different times: 1 min before the first stimulation train and 1 min after the last stimulation train. With repeated stimulation trains, PVN MNCs shifted from a predominantly excitatory state before the first stimulation to a more inhibitory state after repeated stimulation trains (Figure 4G). These findings suggest that repeated optogenetic stimulation gradually enhances inhibitory strength in this subset of PVN MNCs, highlighting a differential response in inhibitory versus excitatory synaptic activity in response to repetitive optogenetic stimulation.

### 3.3 Effects of CIH on EPSC, IPSC, and neuronal excitability

#### 3.3.1 CIH increases PVN magnocellular neurons EPSC amplitude but decreases EPSC frequency

**FIGURE 5:**
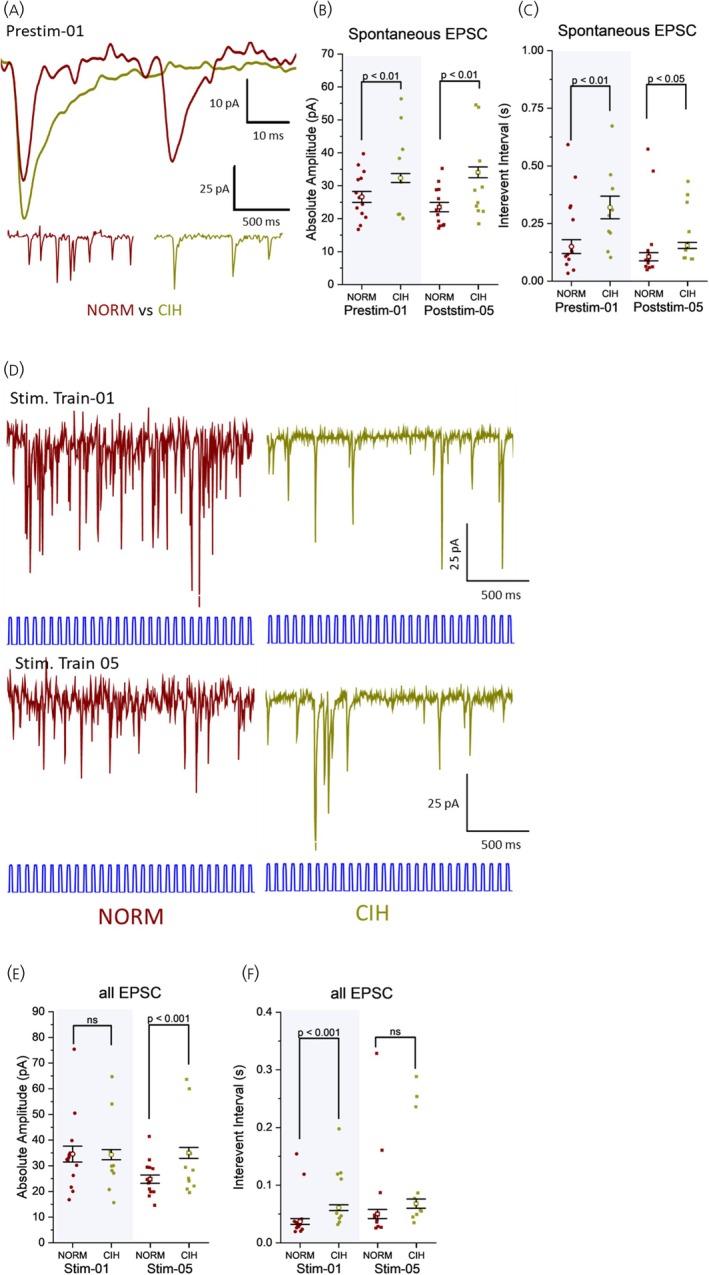
CIH exposure significantly increases the amplitude but decreases the frequency of spontaneous EPSCs (sEPSCs) in PVN MNCs. CIH exposure sustains the increased amplitude but decreases the frequency of all EPSCs during stimulation trains in PVN MNCs. (A) Representative sEPSC recorded from Type I PVN neurons demonstrating the effects of CIH. (B) CIH exposure consistently increased the amplitude of sEPSCs as compared with normoxic controls. (Prestim‐01: *F*(1, 9946) = 6.511, *p* = .008; Poststim‐05: *F*(1, 11,575) = 22.789, *p* < .001). (C) CIH exposure consistently decreased the frequency of sEPSCs (i.e., increased sEPSC IEI) across the recording period (Prestim‐01 (*F*(1, 9939) = 8.93, *p* = .002); Poststim‐05 (*F*(1, 11,572) = 4.070, *p* = .044)). (D) Representative EPSCs recorded from PVN MNCs demonstrating the effects of CIH. (E) During the first stimulation train, EPSC amplitudes were similar in normoxia and CIH, while prior CIH exposure prevented EPSC amplitude decay during the fifth stimulation train (*F*(1, 24,570) = 14.099, *p* < .001). (F) The frequency of EPSCs were lower in CIH‐exposed neurons during the first stimulation (i.e., with higher IEI (*F*(1, 35,495) = 13.475, *p* < .001)); this difference became nonsignificant during the fifth stimulation train (IEI, *F*(1, 24,546) = 0.714, *p* = .374). B, C and E, F: NORM, normoxic, *n* = 13 cells, 8 animals; CIH, chronic intermittent hypoxia‐exposed, *n* = 10 cells, 6 animals. Prestim‐/Stim‐/Poststim‐ = 1‐min prestimulation baseline/stimulation train/1‐min poststimulation recording. GLMM. Data are presented as estimated marginal mean (empty symbols) ± SE, with individual data plots (filled symbols) representing the mean for each neuron.

CIH had a significant impact on spontaneous EPSCs from PVN MNCs irrespective of their optogenetic stimulation response pattern, particularly on their amplitude and frequency. CIH consistently increased the amplitude of spontaneous EPSCs compared with normoxic controls at various time points, including during baseline (prestimulation‐01, Figure 5A,B) and after repeated stimulation trains (poststimulation‐05, Figure 5B). A faster rise time of baseline EPSCs before the first stimulation train and all EPSCs of the first stimulation train was also observed in CIH‐exposed neurons. However, these differences were not apparent during and after the last stimulation train (Figure S6A,B). Prior exposure to CIH consistently led to a significant reduction in spontaneous EPSC frequencies compared with normoxic controls across the recording protocol (Figure 5C).

The effect of CIH exposure on EPSC amplitude during the stimulation period became evident after repeated stimulation trains. Initially, EPSC amplitude was comparable between CIH and normoxic groups (Figure 5D,E). However, while the normoxic group exhibited a decline, CIH‐exposed neurons maintained EPSC amplitude following repeated stimulations (Figure 5D,E). An opposite pattern can be observed in the effects of CIH exposure on the EPSC frequency during stimulation trains. During the first stimulation train, the frequency of EPSCs was lower in CIH‐exposed than in NORM control (Figure 5D,F). In contrast, the fifth stimulation train in CIH neurons elicited EPSCs at a comparable frequency to NORM controls (Figure 5D,F). This indicates that while CIH increases and sustains the strength of excitatory transmission, it reduces the frequency of synaptic events in PVN MNCs.

#### 3.3.2 CIH enhances IPSCs

**FIGURE 6:**
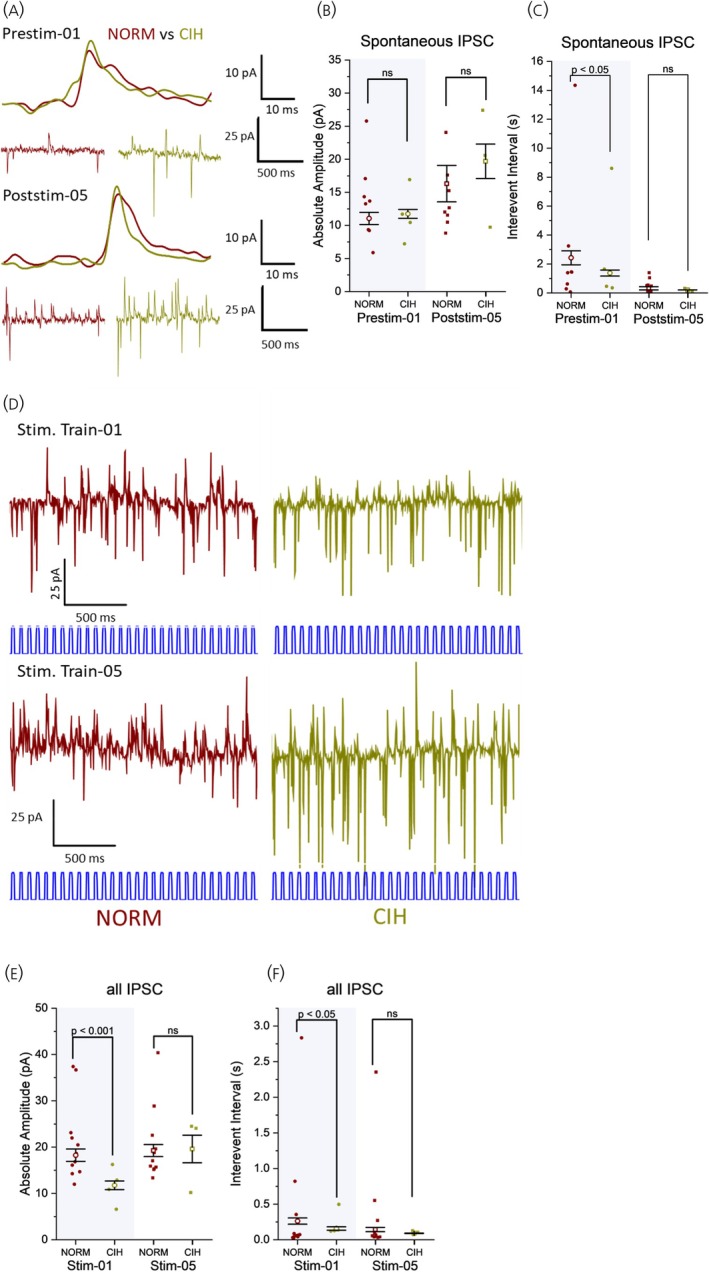
Effects of CIH on spontaneous IPSCs (sIPSCs) and all IPSCs during stimulation train compared with normoxic controls. (A) Representative sIPSCs recorded from PVN MNCs demonstrating the effects of CIH. (B) sIPSC amplitude did not differ between NORM and CIH before the first stimulation train (*F*(1, 1736) = 0.357, *p* = .546); however, a trend for increase in amplitude was observed in CIH neurons compared with NORM by the fifth stimulation (*F*(1, 3607) = 0.774, *p* = .379). (C) Before the first stimulation, spIPSC IEI in CIH was significantly lower than in NORM, indicating a higher frequency (*F*(1,1735) = 5.184, *p* = .045). Following repeated optogenetic stimulations, spIPSC IEI decreased even further, but the difference between NORM and CIH became nonsignificant (Poststim‐05: *F*(1, 3605) = 1.368, *p* = .242). (D) Representative IPSCs recorded from PVN MNCs demonstrating the effects of CIH. (E) The amplitude of IPSCs was initially lower in CIH‐exposed neurons than in NORM but increased after repeated stimulation trains. In contrast, the IPSC amplitude in NORM was already high and remained elevated after repeated stimulation trains. (Stim‐01: *F*(1, 12,250) = 16.653, *p* < .001; Stim‐05: *F*(1, 11,350) = 0.022, *p* = .9161). (F) CIH led to a significantly lower IPSC IEI compared with NORM during the first stimulation train (*F*(1, 12,219) = 4.851, *p* = .041). Repeated optogenetic stimulations reduced IPSC IEI in both groups, eliminating significant differences by the fifth stimulation train (*F*(1, 11,334) = 0.216, *p* = .075). Interpretation of these results is limited by the small sample size, which may affect the robustness and reproducibility of the findings. B, C and E, F: NORM, normoxic, *n* = 11 cells, 8 animals; CIH, chronic intermittent hypoxia‐exposed, *n* = 4 cells, 3 animals. Prestim‐/Stim‐/Poststim‐ = 1‐min prestimulation baseline/stimulation train/1‐min poststimulation recording. GLMM. Data are presented as estimated marginal mean (empty symbols) ± SE, with individual data plots (filled symbols) representing the mean for each neuron.

Prior exposure to CIH had distinct effects on IPSCs compared with EPSCs. Before the first stimulation, baseline sIPSC amplitude was comparable between normoxic and CIH conditions (Figure 6A,B). Although amplitudes remained statistically similar after repeated stimulations, the CIH group exhibited a trend toward increased amplitude. Baseline sIPSC frequency was initially elevated in CIH but became statistically indistinguishable from normoxia as frequency increased in both groups after repeated stimulations (Figure 6A,C).

The first optogenetic stimulation train elicited IPSCs with a lower amplitude and higher frequency in CIH neurons compared with normoxic controls (Figure 6D–F). No significant difference in IPSC amplitude or frequency was detected in the fifth stimulation train between normoxic and CIH groups, reflecting an increase in amplitude in post‐CIH neurons (Figure 6D–F). Collectively, these findings suggest an activity‐dependent strengthening of inhibitory projections to PVN MNCs in CIH‐exposed (*n* = 4 neurons), but not in normoxic MNCs (*n* = 6 neurons). However, the low sample sizes limit the interpretative value of these observations.

### 3.4 CIH facilitates the increase of PVN MNC excitability after optogenetic stimulations

**FIGURE 7:**
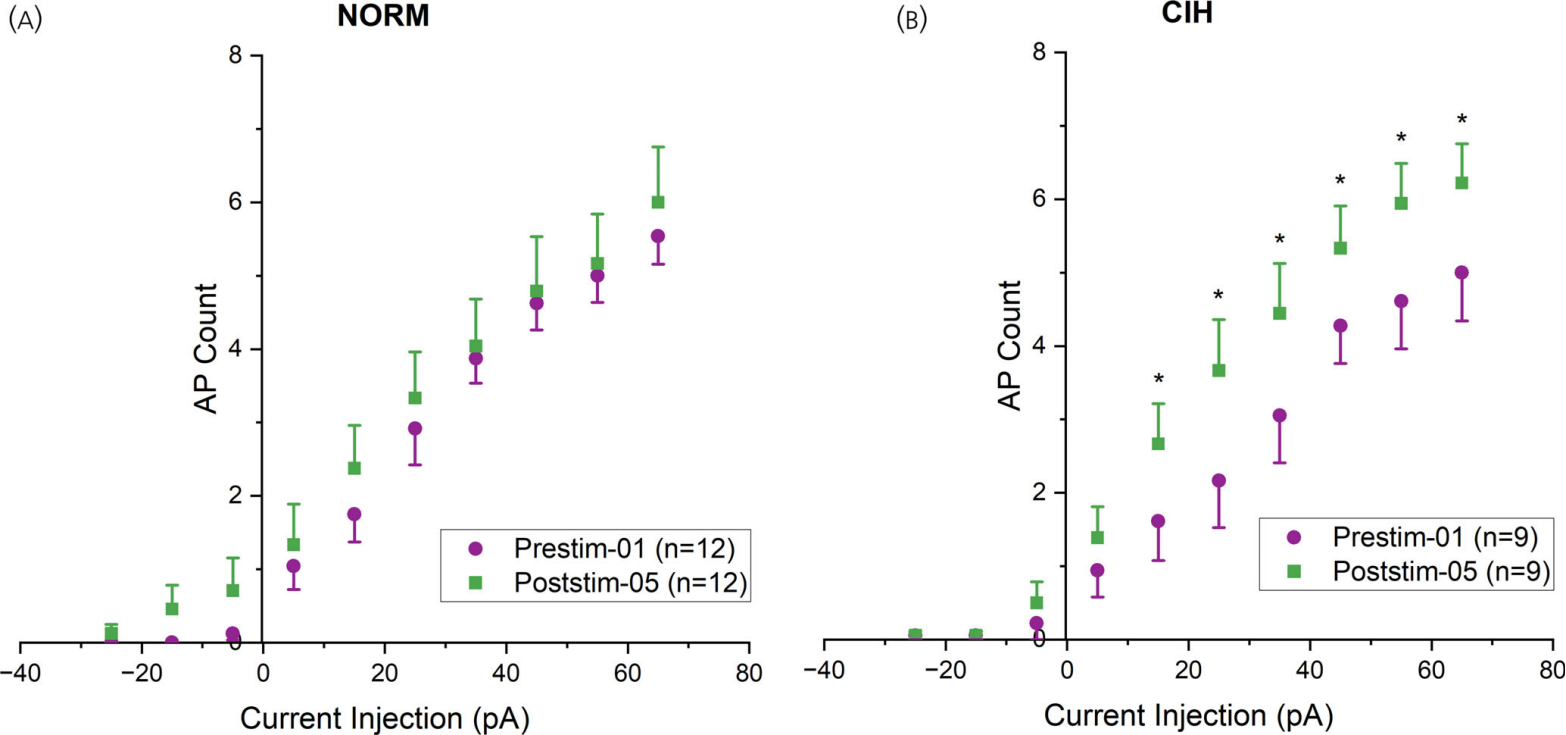
Neuronal excitability before and after repeated stimulations in normoxic and CIH‐exposed PVN MNCs. (A) The relationship between injected depolarizing current and action potential frequency in normoxic MNCs before repeated optogenetic stimulations (empty circles) and after repeated optogenetic stimulations (filled circles). Graded current injections evoked graded increases in AP frequency in CIH‐exposed MNCs similarly between Prestim‐01 and Poststim‐05 (*F*(9, 99) = 0.353, *p* = .95) Two‐way repeated measures ANOVA. NORM, normoxic, *n* = 12 cells, 8 animals. Data are presented as means ± SE. (B) The relationship between injected depolarizing current and action potential frequency in CIH‐exposed MNCs before (empty rhombi) and after repeated optogenetic stimulations (filled rhombi). Graded current injections evoked graded increases in AP frequency in CIH‐exposed MNCs differently between Prestim‐01 and Poststim‐05 (*F*(9, 72) = 3.40, *p* = .0016). Two‐way repeated measures ANOVA. CIH, chronic intermittent hypoxia‐exposed, *n* = 9 cells, 6 animals. Data are presented as means ± SE. *p < .05.

Using whole‐cell patch‐clamp recording in current clamp mode, we found that baseline excitability between NORM and CIH neurons did not differ (Figure S7). Repeated stimulation trains did not alter excitability among PVN MNCs from normoxic controls (Figure 7A). In contrast, repeated optogenetic stimulations in CIH‐exposed neurons increased the number of action potentials triggered by graded injections of depolarizing current (Figure 7B). To determine if there were changes in input resistance, we tested the input resistances of NORM and CIH neurons before and after repeated stimulation trains. A two‐way ANOVA revealed no significant main effects of hypoxia [*F*(1, 32) = 2.68, *p* = .1114] or stimulation [*F*(1, 32) = 0.361, *p* = .5525], and no significant interaction between the two factors [*F*(1, 32) = 2.62, *p* = .1157]. Prior to stimulation, the input resistances were not different (Prestim‐01. NORM 899.6 ± 193.1 MΩ vs. CIH 903 ± 141 MΩ). After repeated stimulation trains, the input resistance did not show significant changes in either NORM or CIH neurons (NORM: Prestim‐01 899.6 ± 193.1 MΩ, Poststim‐05 520.1 ± 99.91 MΩ; CIH: Prestim‐01 903 ± 141 MΩ, Poststim‐05 1077 ± 230 MΩ). These results suggest that the increased excitability observed after repeated stimulations in CIH neurons cannot be attributed to changes in input resistance.

## 4 DISCUSSION

The present study elucidates the response patterns of paraventricular nucleus magnocellular neurons (PVN MNCs) to optogenetic stimulation of the PVN‐projecting MnPO neurons, reports the effects of optogenetic activation of this pathway on excitatory and inhibitory synaptic activity, and examines the impact of chronic intermittent hypoxia (CIH) on synaptic strength. While earlier studies have explored the general activation of various PVN subnuclei under CIH and highlighted the importance of MnPO inputs to the PVN in maintaining CIH‐associated hypertensive features, they have not explicitly addressed the influence of MnPO inputs, CIH, and the interplay between those factors on synaptic activity in PVN MNCs.

Our findings identify distinct response pattern groups among PVN MNCs, revealing differences in EPSC and IPSC profiles that suggest glutamatergic and GABAergic transmission in MnPO‐driven responses. Optogenetic stimulation of MnPO axons at 15 Hz increased spontaneous EPSC frequency without altering amplitude while gradually increasing IPSC frequency and amplitude, shifting some neurons from a predominantly excitatory state to a more inhibitory state. These observations might suggest that the activation of MnPO inputs is sufficient to induce activity‐dependent plasticity in the glutamatergic and GABAergic synapses of PVN MNCs. CIH significantly enhanced the amplitude of EPSCs while reducing their frequency. Concurrently, CIH strengthened inhibitory transmission by increasing both the amplitude and frequency of IPSCs, indicating the presence of heterosynaptic plasticity within the system. Following optogenetic stimulation delivered to neurons after CIH exposure, the intrinsic excitability of PVN MNCs increased compared with before stimulation, indicating that CIH serves as a priming factor for metaplasticity manifested by increased neuronal excitability. These results provide valuable insights into synaptic adaptations and intrinsic plasticity associated with CIH and its influence on MnPO inputs to PVN MNCs.

The MnPO–PVN projection plays a key role in various physiological processes.^4^, ^20^ PVN‐projecting MnPO neurons are responsive to circulating Ang II, hypertonic saline, and changes in blood pressure.^18^ Chemogenetic inhibition of this MnPO–PVN pathway attenuates vasopressin release in response to Ang II or hypertonic saline and significantly decreases Fos staining in magnocellular neurosecretory cells (MNCs) associated with Ang II.^64^ Figure 3B illustrates MnPO–PVN MNC connectivity, investigated using an intersectional optogenetic approach where PVN‐projecting MnPO neurons were activated via a retrograde AAV vector expressing hSyn.Cre in the PVN and a Cre‐dependent channelrhodopsin‐2 in the MnPO. The hSyn promoter was selected for its ability to target both glutamatergic and GABAergic projections, potentially recruiting angiotensin‐responsive MnPO neurons projecting to the PVN.^20^, ^40^, ^64^ Optogenetic stimulation of the MnPO–PVN pathway primarily evoked EPSCs in patched PVN MNCs, though IPSCs were also elicited in some neurons.

In some MNCs, optogenetic stimulation evoked both IPSCs and EPSCs, with IPSCs exhibiting shorter latency and lower jitter than EPSCs. This could be explained by differences in axon diameter or myelination between GABAergic and glutamatergic MnPO–PVN projections, though this hypothesis is unlikely given that IPSC latency and jitter in the IPSC/EPSC sequence pattern were comparable to EPSCs in other response groups. Alternatively, blue light stimulation may have simultaneously activated both inhibitory and excitatory MnPO–PVN neurons due to the non‐specific expression driven by the hSyn promoter.^65^, ^66^ Additionally, the observed delay between IPSCs and EPSCs, along with the slower EPSC latency and higher jitter in neurons with the IPSC/EPSC sequence, may result from a simultaneous activation of a monosynaptic inhibitory projection, together with polysynaptic excitatory projections. The excitatory limb of the pathway begins with a neuron originating in the MnPO, which synapses onto a second excitatory neuron located within the PVN (Figure 3B). This “inhibition‐first” pattern resembles the circuitry of Purkinje cells, where mossy fiber stimulation recruits granule cells that initiate inhibition before excitation, thus influencing synaptic integration and decreasing the likelihood of action potential generation in Purkinje cells.^67^, ^68^, ^69^, ^70^ Meanwhile, in the EPSC/IPSC sequence pattern, longer‐latency IPSCs that are less time‐locked to the stimulation pulse may indicate the delayed recruitment of inhibitory neurons that project to PVN MNCs following the initial activation of monosynaptic excitatory inputs directly targeting these cells. The former could reside in the perinuclear zone of the anterior hypothalamic area that surrounds the PVN (Figure 3B).^71^, ^72^ Thus, in our study, optogenetic photostimulation of the MnPO–PVN pathway appears to consist of direct (monosynaptic) excitatory or inhibitory projections as well as indirect and polysynaptic connections. It is worth mentioning that while in our study, spontaneous and evoked IPSCs were recorded concurrently with EPSCs at a holding potential of around −54 mV, consistent with a previous report,^73^ the slower kinetics of IPSCs relative to EPSCs suggest that these inhibitory currents originate distally within the dendrites and are substantially filtered as they propagate toward the soma.

The responses of MNCs to optogenetic stimulation of the MnPO–PVN pathway demonstrated variability during the stimulus train (Figure 5D–F). The variability of these responses could be related to synaptic reliability and response failure in the context of repeated stimulation. The occurrence of spontaneous PSCs during stimulation could also have contributed to the variability. For this reason, we analyzed the effects of optogenetic stimulation on currents recorded before and after the stimulation to determine the effects of MnPO–PVN stimulation on spontaneous postsynaptic activity. This is similar to the approach used by Iremonger and Bains^74^ with electrical stimulation. Alternatively, when we compared the effects of CIH and repeated stimulation during the stimulation trains, all of the PSCs were included due to the variability, as previously discussed, and the occurrence of possible spontaneous PSCs.

We employed a 15 Hz stimulation protocol (1 min), as used by Frazier et al.,^20^ which approximates the firing rate of MnPO neurons following Ang II activation.^18^ The first optogenetic stimulation train transiently increased sEPSC frequency in MNCs, which returned to near‐baseline levels within 5 min. Additionally, there was a trend toward increased sIPSC frequency after the initial stimulation train. These findings align with previous reports demonstrating that brief stimulations at frequencies as low as 10 Hz can enhance sEPSC frequency via monosynaptic asynchronous glutamate release^74^, ^75^ and increase sIPSC frequency through GABAergic postsynaptic currents.^76^ The absence of poststimulatory changes in EPSC and IPSC amplitudes suggests that amplitude modulation requires a higher stimulation frequency. Electrical high‐frequency stimulation (HFS) at 50–100 Hz has been shown to sustain potentiation for over 15 min.^77^, ^78^, ^79^, ^80^, ^81^ Our findings, which reveal a transient increase in spontaneous EPSC frequency that declines over several minutes, along with a monosynaptic EPSC latency and jitter profile, are consistent with prior observations and indicate that our optogenetic stimulation protocol effectively induces presynaptic potentiation in the MnPO–PVN pathway.

Our optogenetic stimulation paradigm (1 min of stimulation periods separated by 5 min) mimics acute intermittent hypoxia (AIH), which induces neuroplasticity in phrenic and sympathetic nerve activity.^56^, ^57^, ^82^ AIH‐driven sympathetic long‐term facilitation (sLTF) involves circulating Ang II and the PVN.^55^, ^56^, ^57^ Similarly, intermittent optogenetic stimulation of the NTS produces a sympathetic response.^60^ In our study, repeated stimulation trains in PVN MNCs increased sEPSC frequency, though the effect diminished over time, while gradually enhancing both sIPSC amplitude and frequency.

The increase in IPSC frequency suggests increased GABA release that could be due to non‐synaptic mechanisms acting on presynaptic terminals, possibly from local GABAergic interneurons (Figure 3B). Examples of such mechanisms could include NMDA receptor‐driven nitric oxide (NO) release from AVP MNCs during early CIH pathophysiology.^83^, ^84^, ^85^ Increased IPSC frequency may also result from locally released vasopressin, which enhances sIPSC frequency through a predominantly presynaptic mechanism.^86^ Additionally, in CIH‐exposed rats, Ang II type 2 receptor activation in peri‐PVN GABAergic neurons may strengthen inhibitory transmission onto AVP neurons.^87^ The progressive increase in IPSC amplitude may result from glutamate–GABA strengthening, where synaptic glutamate uptake enhances presynaptic GABA supply, leading to greater inhibition in PVN MNCs.^88^ These findings collectively suggest multiple mechanisms by which repeated optogenetic stimulation may enhance GABAergic inhibition of PVN MNCs, including through the recruitment of local inhibitory neurons (Figure 3B).

The activity of PVN neurons depends on the balance between excitatory glutamate and inhibitory GABA.^73^, ^89^ Potapenko et al. found that while GABAergic strength exceeded glutamatergic strength in both sham and heart failure rats, heart failure shifted the balance toward excitation.^73^ In our study, repeated optogenetic stimulation increased the IPSC/EPSC ratio, shifting some PVN MNCs from an excitatory to a more inhibitory state. This suggests a differential adjustment in inhibitory versus excitatory synaptic activity in response to repeated stimulation. Such priming could serve to protect against a glutamate surge resulting from the activation of MnPO and possibly other visceral afferent inputs (not examined).

In a study using Cre recombinase to target ChR2 expression in Agtr1a‐expressing MnPO neurons, optogenetic stimulation increased systolic blood pressure, an effect blocked by a vasopressin receptor antagonist.^20^ Increased activity of PVN MNCs in response to MnPO optogenetic stimulation might evoke the dendritic release of AVP and OT from MNCs^46^, ^90^, ^91^ and thus affect neighboring neurons such as V1aRs expressing PVN preautonomic parvocellular neurons.^46^ Vasopressin levels sufficient to normalize ESPSC amplitude^92^ evoke sufficient dendritic release of vasopressin to increase sympathetic nerve activity as well.^46^ Direct synaptic activation of preautonomic neurons by PVN‐projecting MnPO neurons, along with AVP dendritic release, may account for the increased blood pressure in CIH‐exposed animals.

A single episode of acute hypoxia can result in a rise in the level of AVP in the plasma of many species.^93^, ^94^, ^95^, ^96^ Interestingly, stimulation of carotid body chemoreceptors has no effect on MNCs in PVN,^97^ but it increases the activity of SON neurons through a pathway involving the preoptic area.^98^ However, since acute hypoxia causes a decrease in blood pressure,^94^, ^96^ and because MnPO neurons projecting to PVN respond to alterations in baroreceptor stimulation,^18^ integrative processing by the MnPO–PVN pathway likely participates in physiological and perhaps pathophysiological responses to acute hypoxia. Given that the MnPO–PVN pathway is also known to contribute to the sustained increase in blood pressure associated with CIH,^39^ it appears that the MnPO–PVN pathway is necessary for the development of the sustained hypertension resulting from sleep apnea/CIH in rats.^99^

CIH, acute hypoxia, and AIH are associated with increased expression of FosB and c‐Fos in PVN MNCs.^39^, ^55^, ^100^, ^101^ Expression of these Ca^2+^‐dependent transcription factors serves as markers of neuronal activity and is involved in the regulation of genes that mediate synaptic changes. However, the precise role of each marker in synaptic activity differs across various brain regions. In hippocampal CA1 neurons, FosB modulation reduces EPSC amplitude without affecting frequency and affects their intrinsic excitability,^102^ while Fos expression bidirectionally alters IPSC amplitude, influencing inhibition from distinct interneuron types.^49^ In MnPO neurons, blocking the transcriptional activity of FosB prevents the development of sustained CIH hypertension.^25^ However, the effects of acute and chronic intermittent hypoxia on synaptic activity of PVN MNCs, presumably mediated through the expression of immediate early genes, are largely unknown.

In our study, CIH increased the amplitude of sEPSCs and EPSCs recorded during stimulation but decreased EPSC frequency in PVN MNCs, while repeated MnPO–PVN stimulation enhanced inhibition by increasing the amplitude and frequency of sIPSCs and IPSCs recorded during high‐frequency stimulation. CIH also increased the proportion of neurons exhibiting only EPSC responses. In contrast, CIH in the NTS reduced stimulation‐evoked EPSC amplitude via presynaptic mechanisms but increased the frequency of miniature and asynchronous EPSCs, thereby balancing spontaneous activity with reduced evoked responses.^103^, ^104^ Despite differing directions, these synaptic adaptations in the PVN and NTS likely serve as homeostatic plasticity to maintain neuronal stability.^105^

Repeated optogenetic stimulation of the MnPO–PVN pathway did not alter PVN MNC excitability under normoxic conditions but enhanced it after CIH exposure. This suggests CIH primes the pathway, making low‐frequency stimulation sufficient to increase excitability.^106^ In our preparation, neither lower‐frequency stimulation alone nor CIH without stimulation affected excitability. This aligns with in vivo findings that AIH in nephrectomized rats or Ang II without AIH fails to induce sympathetic long‐term facilitation, emphasizing the need for both circuitry activation and modulatory factors.^56^ A limitation of this study is the lack of phenotypic characterization of the recorded neurons, preventing the determination of AVP or OT expression. Consequently, it remains unclear whether distinct phenotypes exhibit differential response patterns.

In summary, this study explored the role of the MnPO in regulating the activity of PVN MNCs, with a particular focus on the effects of CIH. Using optogenetic methods, acute activation of MnPO neurons projecting to the PVN was found to affect excitatory and inhibitory synaptic potentials differentially. CIH enhanced synaptic excitatory strength and increased the intrinsic excitability of PVN MNCs while simultaneously attenuating overall synaptic activities. The predominant response to optogenetic stimulation of the MnPO–PVN pathway was synaptic excitation. However, in CIH‐exposed neurons, this appears to be counterbalanced by an increase in inhibitory tone in a significant fraction of PVN MNCs. These parallel increases in synaptic inhibition may offset the heightened excitability, possibly explaining the lack of significant changes in neurohypophyseal hormone release observed in prolonged exposure to CIH.

## 5 PERSPECTIVE AND SIGNIFICANCE

The MnPO serves as a critical regulatory center that modulates thirst, cardiovascular functions, and neuroendocrine activity. Its connection to the PVN plays a pivotal role in sustaining the hypertension associated with CIH. Selective optogenetic stimulation of the MnPO–PVN pathway elicits early onset and transient synaptic plasticity, characterized by an increase in synaptic activity that persists only briefly beyond the stimulation period. Following CIH exposure, the response to MnPO–PVN stimulation appears to reflect homeostatic plasticity. While CIH‐induced changes in neuronal excitability, repeated stimulation of the MnPO–PVN pathway also increased inhibitory input. These findings offer new insights into mechanisms that may limit the involvement of PVN MNCs and vasopressin release in the development of CIH‐associated hypertension, as well as in the broader context of clinical sleep apnea. These adaptations may be critical for body fluid homeostasis by pituitary AVP secretion in the face of heightened MnPO synaptic drive to MNCs.

## AUTHOR CONTRIBUTIONS

**Obed T. Paundralingga:** Conceptualization; investigation; methodology; formal analysis; writing – original draft; data curation; writing – review and editing. **Shuping Jia:** Investigation; methodology; validation; formal analysis; writing – review and editing; data curation. **George E. Farmer Jr:** Validation; methodology; writing – review and editing; supervision; conceptualization. **Glenn M. Toney:** Funding acquisition; writing – review and editing; project administration; supervision. **J. Thomas Cunningham:** Conceptualization; writing – review and editing; project administration; resources; supervision; data curation; software; funding acquisition; formal analysis; validation.

## CONFLICT OF INTEREST STATEMENT

The authors have nothing to disclose.

## Supporting information


**Figure S1.** The EPSCs were inhibited by CNQX (10 μM, Tocris, Minneapolis, MN) and the IPSCs were inhibited by Gabazine (25 μM, Tocris, Minneapolis, MN) respectively. Evoked EPSC absolute amplitude before CNQX 51.7 ± 11.2 pA, after CNQX 0.0 pA. *t* ^4^ = 4.63, *p* = .009. Evoked IPSC amplitude before Gabazine 13.8 ± 1.16 pA, after Gabazine 0.0 pA. *t* ^4^ = 11.9, *p* = .0003. Paired *t*‐test. *n* = 5 cells, 3 animals.

**Figure S2.** Absolute amplitudes and interevent intervals of both spontaneous and all stimulatory EPSCs were compared across neurons exhibiting distinct response patterns. No significant differences were observed between response groups during prestim‐01 and prestim‐05, in terms of spontaneous EPSC amplitude and frequency (Figure S1A,B), or all EPSC amplitude and frequency during stimulation (Figure S1C,D). (A) Amplitude sEPSC: Prestim 01 *F*(2, 7419) = 1.175, *p* = .309; Prestim 05 *F*(2, 5276) = 0.660, *p* = .517. (B) IEI sEPSC: Prestim 01 (2, 7413) = 0.423, *p* = .655; Prestim 05 (2, 5276) = 1.362, *p* = .256. (C) Amplitude EPSC: Stim 01 *F*(2, 25,218) = 3.667, *p* = .026; Stim 05 *F*(2, 16,067) = 0.667, *p* = .508. (D) IEI EPSC: Stim 01 (2, 25,167) = 0.035, *p* = .965; Stim 05 (2, 16,052) = 0.414, *p* = .661. All comparisons: IEI = inter‐event interval (i.e., 1/frequency). Prestim‐01/05 = 1 min prestimulation baseline 01/05. Stim‐01/05 = stimulation train 01/05. GLMM. Normoxic IPSC/EPSC *n* = 5 cells, 4 animals, EPSC/IPSC *n* = 5 cells, 4 animals, EPSC *n* = 3 cells, 3 animals. Data are expressed as estimated marginal mean ± SE.

**Figure S3.** IPSC/EPSC sequence response and EPSC/IPSC sequence response groups exhibited differences in spontaneous and all stimulatory IPSC frequencies in normoxic controls. (A) Amplitude sIPSC: Prestim 01 *F*(1, 1390) = 2.803, *p* = .094; Prestim 05 *F*(1, 1129) = 1.129, *p* = .288. (B) IEI sIPSC: Prestim 01 *F*(1, 1389) = 6.646, *p* = .010; Prestim 05 *F*(1, 1687) = 0.500, *p* = .480. (C) Amplitude IPSC Stim 01 *F*(1, 10,703) = 0.142, *p* = .707; Stim 05 *F*(1, 9237) = 0.388, *p* = .533. (D) IEI IPSC: Stim 01 *F*(1, 10,672) = 6.304, *p* = .012; Stim 05 *F*(1, 9226) = 7.111, *p* = .008. All comparisons: IEI = inter‐event interval (i.e., 1/frequency). Prestim‐01/05 = 1 min prestimulation baseline 01/05. Stim‐01/05 = stimulation train 01/05. GLMM. Normoxic IPSC/EPSC *n* = 5 cells, 4 animals, EPSC/IPSC *n* = 5 cells, 4 animals. Data are expressed as estimated marginal mean ± SE.

**Figure S4.** Immediate and progressive effect of optogenetic stimulation on sEPSC amplitude and interevent interval. (A) Amplitude Pre‐ vs. poststim 2 *F*(1, 26,886) = 0.065, *p* = .799; Pre‐ vs. poststim 3 *F*(1, 25,020) = 0.009, *p* = .923; Pre‐ vs. poststim 4, *F*(1, 23,140) = 0.051, *p* = .822; Pre‐ vs. poststim 5, *F*(1, 19,604) = 0.001, *p* = .975. Amplitude Prestim1 vs. poststim 2 *F*(1, 26,886) = 0.002, *p* = .968; vs. poststim 3 *F*(1, 24,885) = 0.165, *p* = .685; vs. poststim 4 *F*(1, 23,927) = 0.609, *p* = .435; vs. poststim 5 *F*(1, 21,387) = 0.906, *p* = .341. (B) IEI Pre‐ vs. poststim 2 *F*(1, 27,268) = 7.479, *p* = .006; Pre‐ vs. poststim 3 *F*(1, 25,017) = 6.801, *p* = .009; Pre‐ vs. poststim 4, *F*(1, 23,140) = 7.396, *p* = .007; Pre‐ vs. poststim 5, *F*(1, 19,600) = 3.537, *p* = .060. IEI Prestim1 vs. poststim 2 *F*(1, 26,878) = 8.647, *p* = .003; vs. poststim 3 *F*(1, 24,875) = 7.162, *p* = .007; vs. poststim 4, *F*(1, 23,920) = 6.841, *p* = .009; vs. poststim 5, *F*(1, 21,377) = 4.459, *p* = .035. All comparisons: IEI = inter‐event interval (i.e., 1/frequency). Prestim‐01 to ‐05 = 1 min prestimulation baseline 01 to 05. Poststim‐01 to 05 = 1‐min poststimulation 01 to 05. GLMM. *n* = 23 cells, 14 animals. Data are expressed as estimated marginal mean ± SE.

**Figure S5.** Immediate and progressive effect of optogenetic stimulation on sIPSC amplitude and interevent interval. (A) Amplitude Pre‐ vs. poststim 2 *F*(1, 5041) = 0.044, *p* = .834; Pre‐ vs. poststim 3 *F*(1, 5009) = 0.084, *p* = .772; Pre‐ vs. poststim 4, *F*(1, 6004) = 0.248, *p* = .618; Pre‐ vs. poststim 5, *F*(1, 6242) = 0.029, *p* = .865. Amplitude Prestim1 vs. poststim 1 *F*(1, 4860) = 0.431, *p* = .512; vs. poststim 2 *F*(1, 4743) = 0.566, *p* = .452; vs. poststim 3 *F*(1, 4840) = 0.991, *p* = .320; vs. poststim 4 *F*(1, 5250) = 2.287, *p* = .130; vs. poststim 5 *F*(1, 5174) = 2.369, *p* = .124. (B) IEI Pre‐ vs. poststim 2, *F*(1, 5038) = 2.494, *p* = .114; Pre‐ vs. poststim 3, *F*(1, 5008) = 5.638, *p* = .018; Pre‐ vs. poststim 4, *F*(1, 6004) = 1.877, *p* = .171; Pre‐ vs. poststim 5, *F*(1, 6240) = 1.369, *p* = .242. IEI Pre‐ vs. poststim 1 *F*(1, 4855) = 2.193, *p* = .139; Pre‐ vs. poststim 2, *F*(1, 4739) = 5.577, *p* = .018; Pre‐ vs. poststim 3, *F*(1, 4838) = 8.433, *p* = .004; Pre‐ vs. poststim 4, *F*(1, 5249) = 10.393, *p* = .001; Pre‐ vs. poststim 5, *F*(1, 5171) = 11.815, *p* < .001. All comparisons: IEI = inter‐event interval (i.e., 1/frequency). Prestim‐01 to 05 = 1 min prestimulation baseline 01 to 05. Poststim‐01 to ‐05 = 1‐min poststimulation 01 to 05. GLMM. *n* = 15 cells, 11 animals. Data are expressed as estimated marginal mean ± SE.

**Figure S6.** The effects of CIH exposure and optogenetic stimulations on EPSC rise time. (A) Prestim 01 NORM vs. CIH *F*(1, 9804) = 6.315, *p* = .012. Poststim 05 NORM vs. CIH *F*(1, 11,575) = 1.598, *p* = .206. (B) Stim 01 NORM vs. CIH *F*(1, 35,391) = 4.767, *p* = .029. Stim 05 NORM vs. CIH *F*(1, 25,457) = 2.280, *p* = .131. All comparisons: IEI = inter‐event interval (i.e., 1/frequency). Prestim‐01 = 1 min prestimulation baseline 01 to 05. Poststim‐05 = 1‐min poststimulation 05. Stim‐01/05 = stimulation train 01/05. GLMM. *n* = 23 cells, 14 animals. Data are expressed as estimated marginal mean ± SE.

**Figure S7.** Neuronal excitability before repeated stimulations in normoxic and CIH‐exposed PVN MNCs. The relationship between injected depolarizing current and action potential frequency in normoxic MNCs (maroon circles) and CIH neurons (golden rhombi). Graded current injections evoked graded increases in AP frequency in CIH‐exposed MNCs similarly between NORM and CIH (*F*(9,69) = 1.36, *p* = .225) Two‐way repeated measures ANOVA. NORM, normoxic, *n* = 21 cells, 14 animals. Data are presented as means ± SE.

## Data Availability

The data that support the findings of this study are available from the corresponding author upon reasonable request.
