# Supplementary figures and images for: Optogenetic stimulation of the median preoptic nucleus: Effects on hypothalamic paraventricular nucleus magnocellular neurons after chronic intermittent hypoxia exposure
Source: J Neuroendocrinol. 2025 Oct 19;38(1):e70100. doi: 10.1111/jne.70100 (PMC12710162; doi:10.1111/jne.70100)

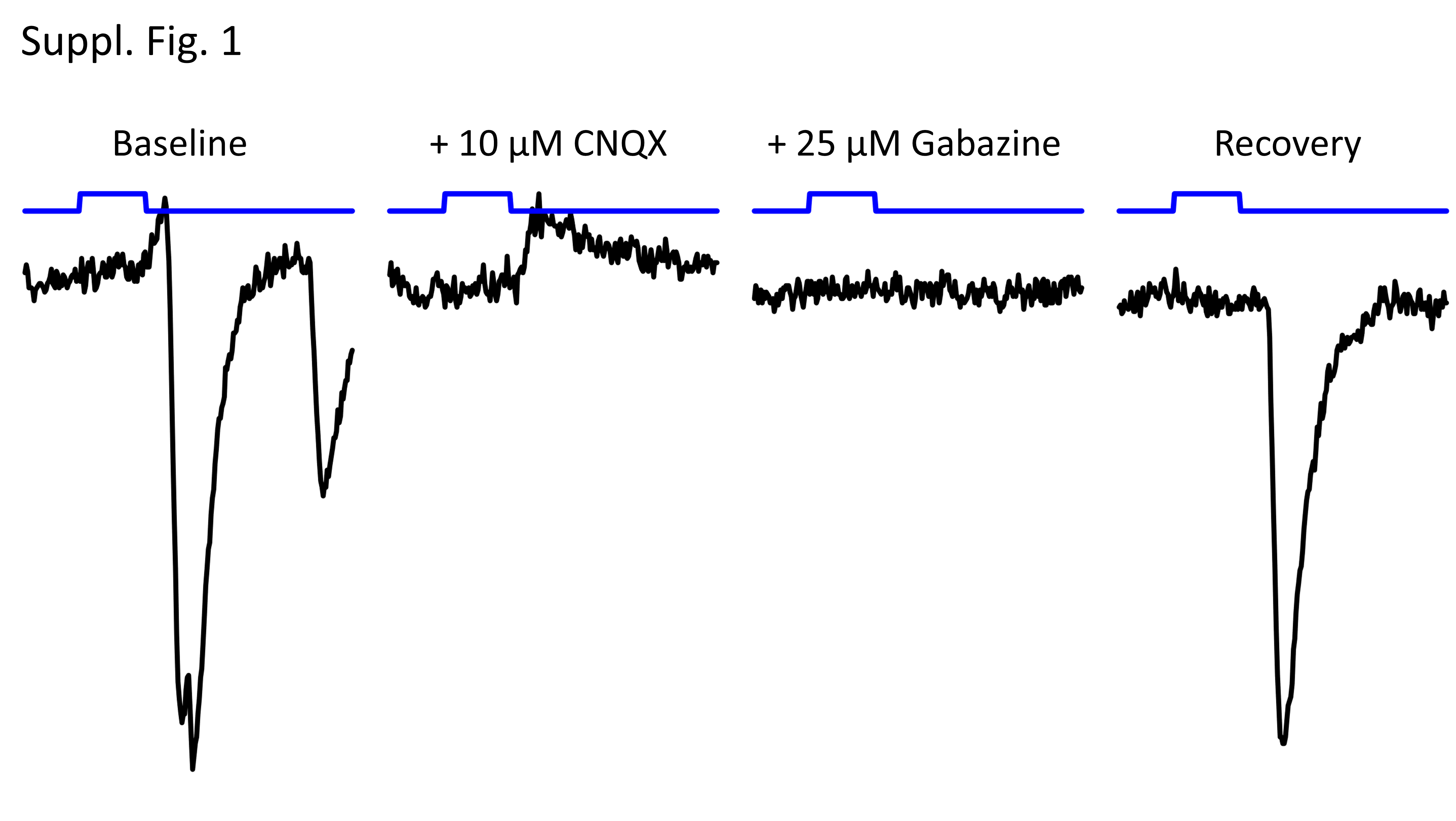

Supplement: Supplementary file 1 — Figure S1. The EPSCs were inhibited by CNQX (10 μM, Tocris, Minneapolis, MN) and the IPSCs were inhibited by Gabazine (25 μM, Tocris, Minneapolis, MN) respectively. Evoked EPSC absolute amplitude before CNQX 51.7 ± 11.2 pA, after CNQX 0.0 pA. t 4 = 4.63, p = .009. Evoked IPSC amplitude before Gabazine 13.8 ± 1.16 pA, after Gabazine 0.0 pA. t 4 = 11.9, p = .0003. Paired t‐test. n = 5 cells, 3 animals. [file JNE-38-e70100-s003.tif]

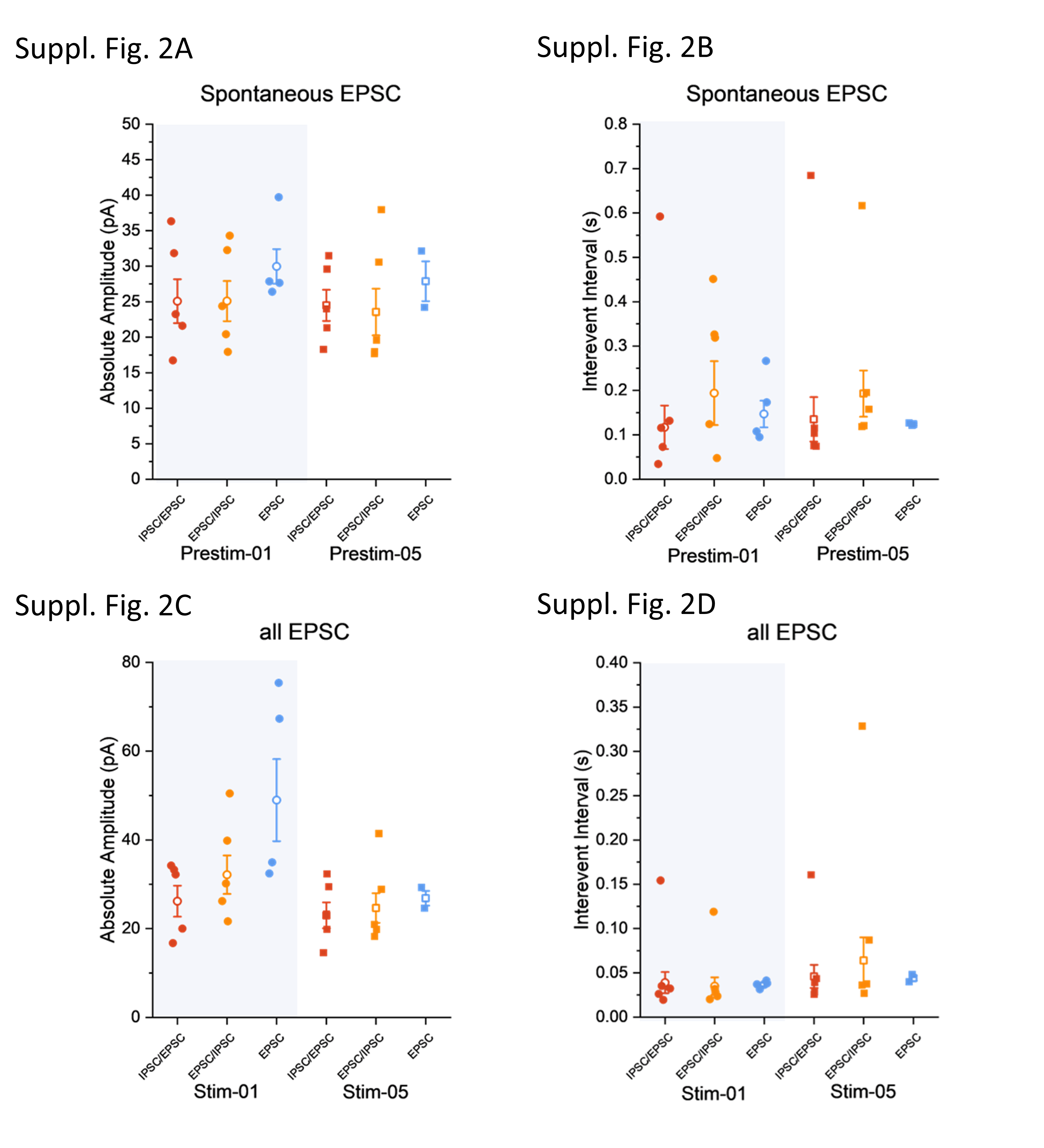

Supplement: Supplementary file 2 — Figure S2. Absolute amplitudes and interevent intervals of both spontaneous and all stimulatory EPSCs were compared across neurons exhibiting distinct response patterns. No significant differences were observed between response groups during prestim‐01 and prestim‐05, in terms of spontaneous EPSC amplitude and frequency (Figure S1A,B), or all EPSC amplitude and frequency during stimulation (Figure S1C,D). (A) Amplitude sEPSC: Prestim 01 F(2, 7419) = 1.175, p = .309; Prestim 05 F(2, 5276) = 0.660, p = .517. (B) IEI sEPSC: Prestim 01 (2, 7413) = 0.423, p = .655; Prestim 05 (2, 5276) = 1.362, p = .256. (C) Amplitude EPSC: Stim 01 F(2, 25,218) = 3.667, p = .026; Stim 05 F(2, 16,067) = 0.667, p = .508. (D) IEI EPSC: Stim 01 (2, 25,167) = 0.035, p = .965; Stim 05 (2, 16,052) = 0.414, p = .661. All comparisons: IEI = inter‐event interval (i.e., 1/frequency). Prestim‐01/05 = 1 min prestimulation baseline 01/05. Stim‐01/05 = stimulation train 01/05. GLMM. Normoxic IPSC/EPSC n = 5 cells, 4 animals, EPSC/IPSC n = 5 cells, 4 animals, EPSC n = 3 cells, 3 animals. Data are expressed as estimated marginal mean ± SE. [file JNE-38-e70100-s002.tif]

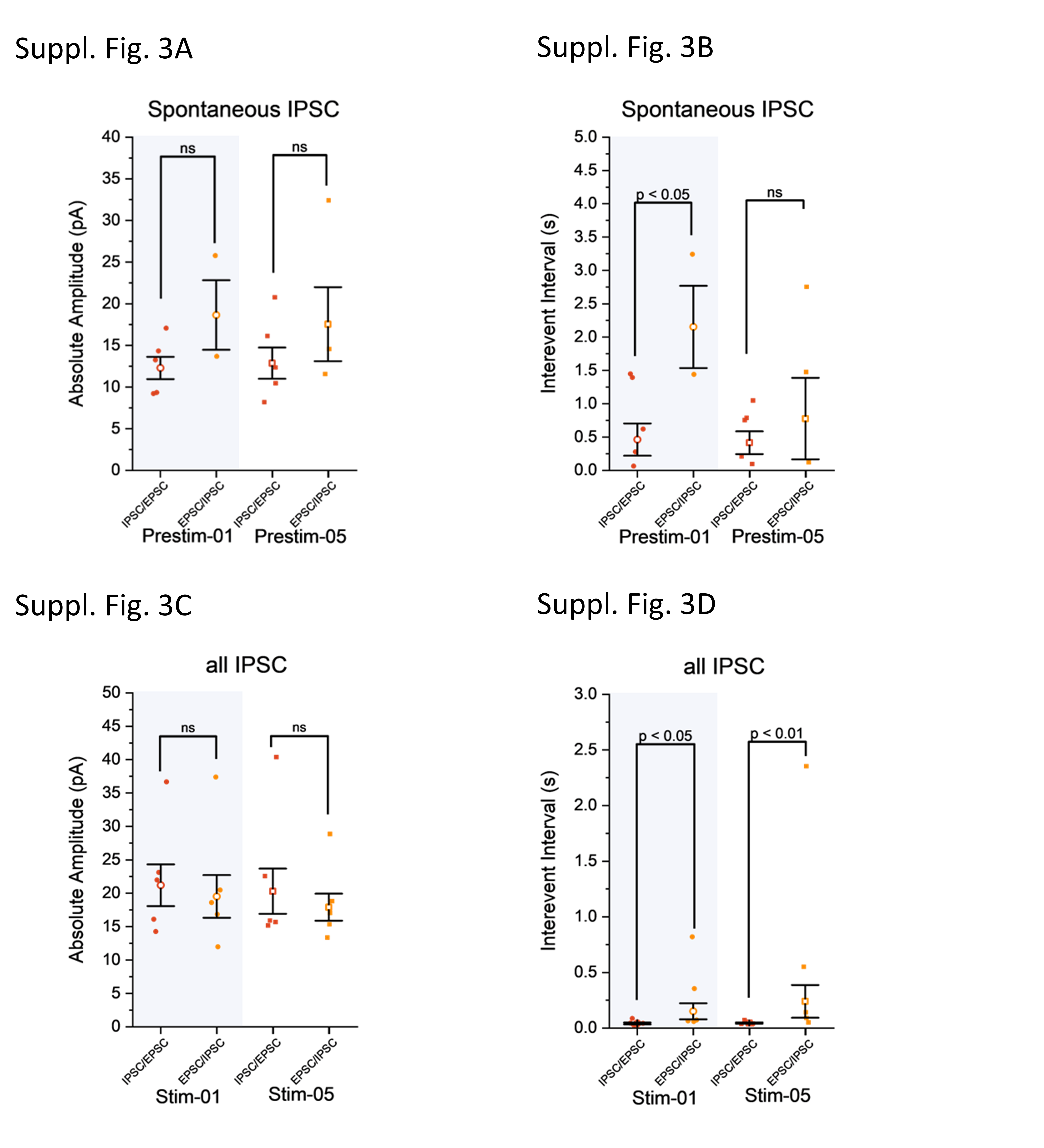

Supplement: Supplementary file 3 — Figure S3. IPSC/EPSC sequence response and EPSC/IPSC sequence response groups exhibited differences in spontaneous and all stimulatory IPSC frequencies in normoxic controls. (A) Amplitude sIPSC: Prestim 01 F(1, 1390) = 2.803, p = .094; Prestim 05 F(1, 1129) = 1.129, p = .288. (B) IEI sIPSC: Prestim 01 F(1, 1389) = 6.646, p = .010; Prestim 05 F(1, 1687) = 0.500, p = .480. (C) Amplitude IPSC Stim 01 F(1, 10,703) = 0.142, p = .707; Stim 05 F(1, 9237) = 0.388, p = .533. (D) IEI IPSC: Stim 01 F(1, 10,672) = 6.304, p = .012; Stim 05 F(1, 9226) = 7.111, p = .008. All comparisons: IEI = inter‐event interval (i.e., 1/frequency). Prestim‐01/05 = 1 min prestimulation baseline 01/05. Stim‐01/05 = stimulation train 01/05. GLMM. Normoxic IPSC/EPSC n = 5 cells, 4 animals, EPSC/IPSC n = 5 cells, 4 animals. Data are expressed as estimated marginal mean ± SE. [file JNE-38-e70100-s005.tif]

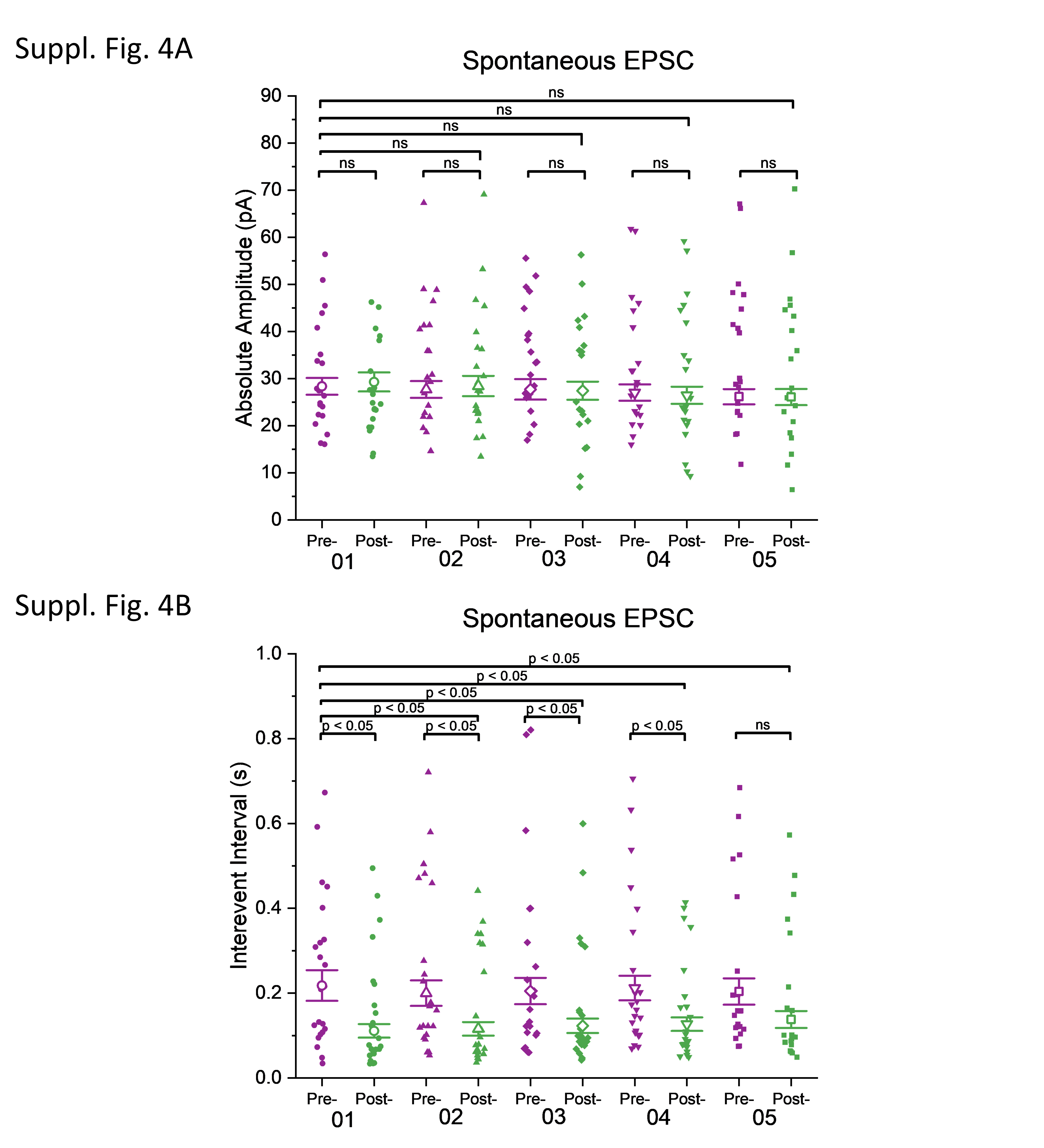

Supplement: Supplementary file 4 — Figure S4. Immediate and progressive effect of optogenetic stimulation on sEPSC amplitude and interevent interval. (A) Amplitude Pre‐ vs. poststim 2 F(1, 26,886) = 0.065, p = .799; Pre‐ vs. poststim 3 F(1, 25,020) = 0.009, p = .923; Pre‐ vs. poststim 4, F(1, 23,140) = 0.051, p = .822; Pre‐ vs. poststim 5, F(1, 19,604) = 0.001, p = .975. Amplitude Prestim1 vs. poststim 2 F(1, 26,886) = 0.002, p = .968; vs. poststim 3 F(1, 24,885) = 0.165, p = .685; vs. poststim 4 F(1, 23,927) = 0.609, p = .435; vs. poststim 5 F(1, 21,387) = 0.906, p = .341. (B) IEI Pre‐ vs. poststim 2 F(1, 27,268) = 7.479, p = .006; Pre‐ vs. poststim 3 F(1, 25,017) = 6.801, p = .009; Pre‐ vs. poststim 4, F(1, 23,140) = 7.396, p = .007; Pre‐ vs. poststim 5, F(1, 19,600) = 3.537, p = .060. IEI Prestim1 vs. poststim 2 F(1, 26,878) = 8.647, p = .003; vs. poststim 3 F(1, 24,875) = 7.162, p = .007; vs. poststim 4, F(1, 23,920) = 6.841, p = .009; vs. poststim 5, F(1, 21,377) = 4.459, p = .035. All comparisons: IEI = inter‐event interval (i.e., 1/frequency). Prestim‐01 to ‐05 = 1 min prestimulation baseline 01 to 05. Poststim‐01 to 05 = 1‐min poststimulation 01 to 05. GLMM. n = 23 cells, 14 animals. Data are expressed as estimated marginal mean ± SE. [file JNE-38-e70100-s007.tif]

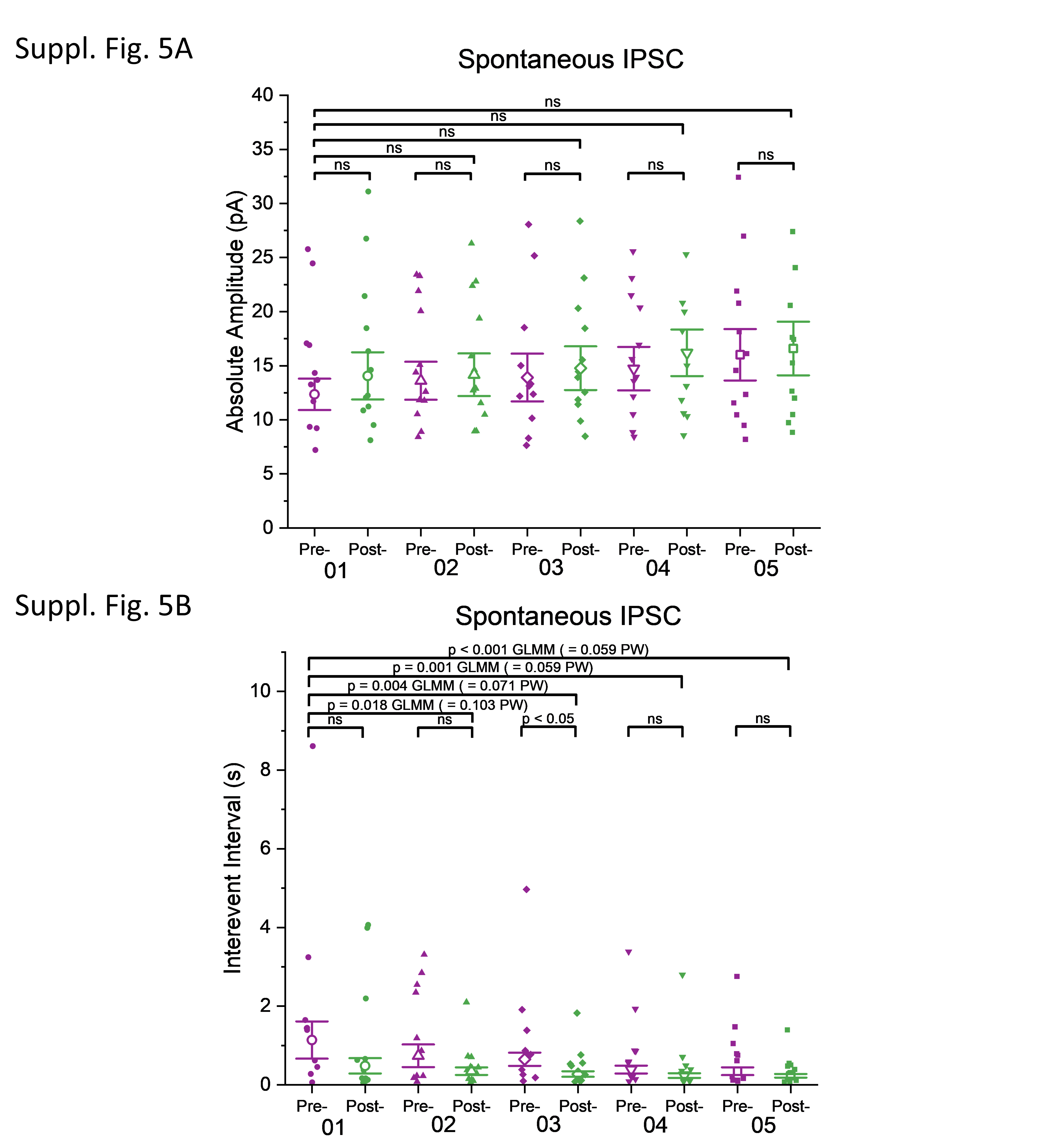

Supplement: Supplementary file 5 — Figure S5. Immediate and progressive effect of optogenetic stimulation on sIPSC amplitude and interevent interval. (A) Amplitude Pre‐ vs. poststim 2 F(1, 5041) = 0.044, p = .834; Pre‐ vs. poststim 3 F(1, 5009) = 0.084, p = .772; Pre‐ vs. poststim 4, F(1, 6004) = 0.248, p = .618; Pre‐ vs. poststim 5, F(1, 6242) = 0.029, p = .865. Amplitude Prestim1 vs. poststim 1 F(1, 4860) = 0.431, p = .512; vs. poststim 2 F(1, 4743) = 0.566, p = .452; vs. poststim 3 F(1, 4840) = 0.991, p = .320; vs. poststim 4 F(1, 5250) = 2.287, p = .130; vs. poststim 5 F(1, 5174) = 2.369, p = .124. (B) IEI Pre‐ vs. poststim 2, F(1, 5038) = 2.494, p = .114; Pre‐ vs. poststim 3, F(1, 5008) = 5.638, p = .018; Pre‐ vs. poststim 4, F(1, 6004) = 1.877, p = .171; Pre‐ vs. poststim 5, F(1, 6240) = 1.369, p = .242. IEI Pre‐ vs. poststim 1 F(1, 4855) = 2.193, p = .139; Pre‐ vs. poststim 2, F(1, 4739) = 5.577, p = .018; Pre‐ vs. poststim 3, F(1, 4838) = 8.433, p = .004; Pre‐ vs. poststim 4, F(1, 5249) = 10.393, p = .001; Pre‐ vs. poststim 5, F(1, 5171) = 11.815, p < .001. All comparisons: IEI = inter‐event interval (i.e., 1/frequency). Prestim‐01 to 05 = 1 min prestimulation baseline 01 to 05. Poststim‐01 to ‐05 = 1‐min poststimulation 01 to 05. GLMM. n = 15 cells, 11 animals. Data are expressed as estimated marginal mean ± SE. [file JNE-38-e70100-s004.tif]

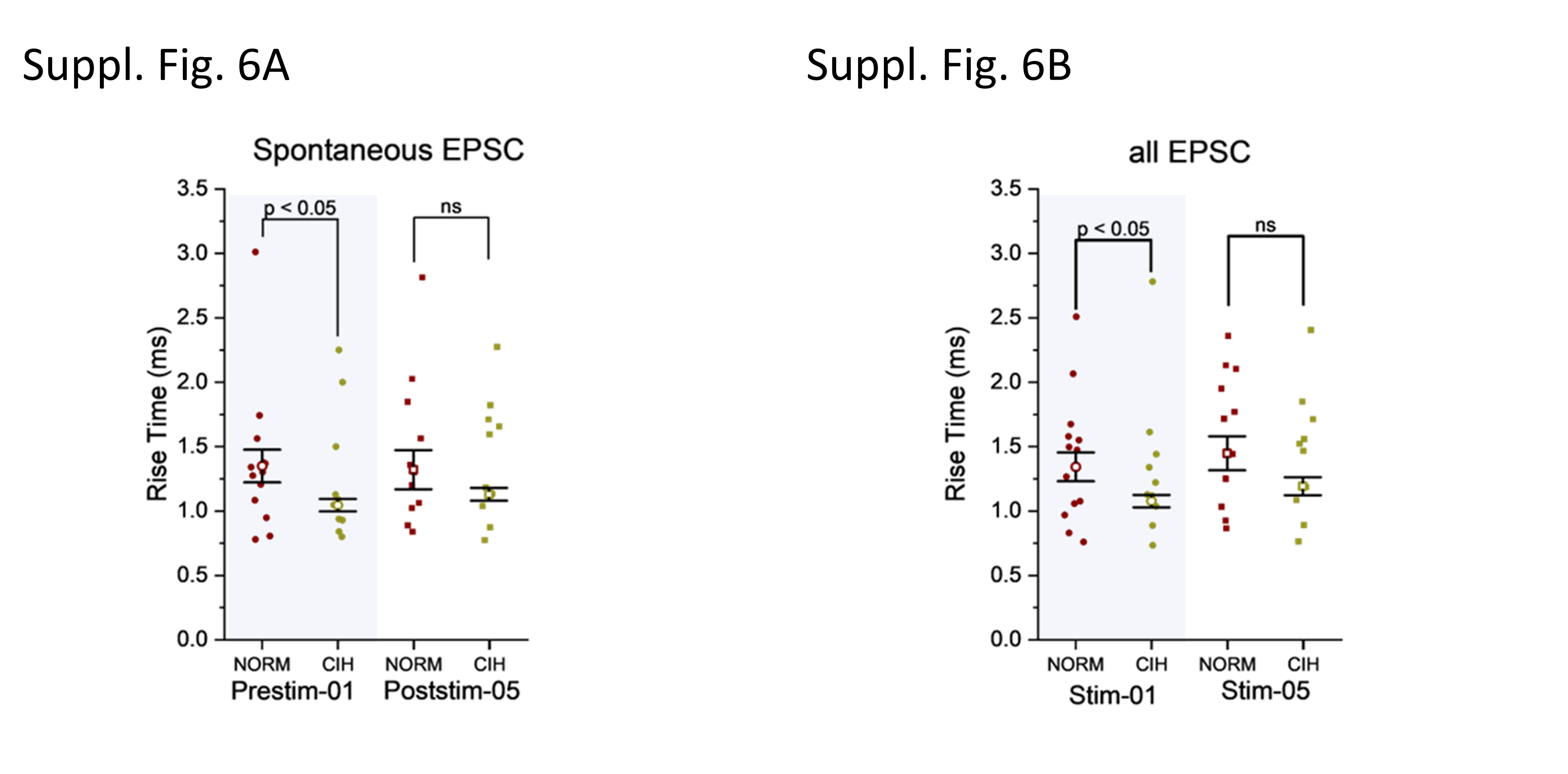

Supplement: Supplementary file 6 — Figure S6. The effects of CIH exposure and optogenetic stimulations on EPSC rise time. (A) Prestim 01 NORM vs. CIH F(1, 9804) = 6.315, p = .012. Poststim 05 NORM vs. CIH F(1, 11,575) = 1.598, p = .206. (B) Stim 01 NORM vs. CIH F(1, 35,391) = 4.767, p = .029. Stim 05 NORM vs. CIH F(1, 25,457) = 2.280, p = .131. All comparisons: IEI = inter‐event interval (i.e., 1/frequency). Prestim‐01 = 1 min prestimulation baseline 01 to 05. Poststim‐05 = 1‐min poststimulation 05. Stim‐01/05 = stimulation train 01/05. GLMM. n = 23 cells, 14 animals. Data are expressed as estimated marginal mean ± SE. [file JNE-38-e70100-s001.tif]

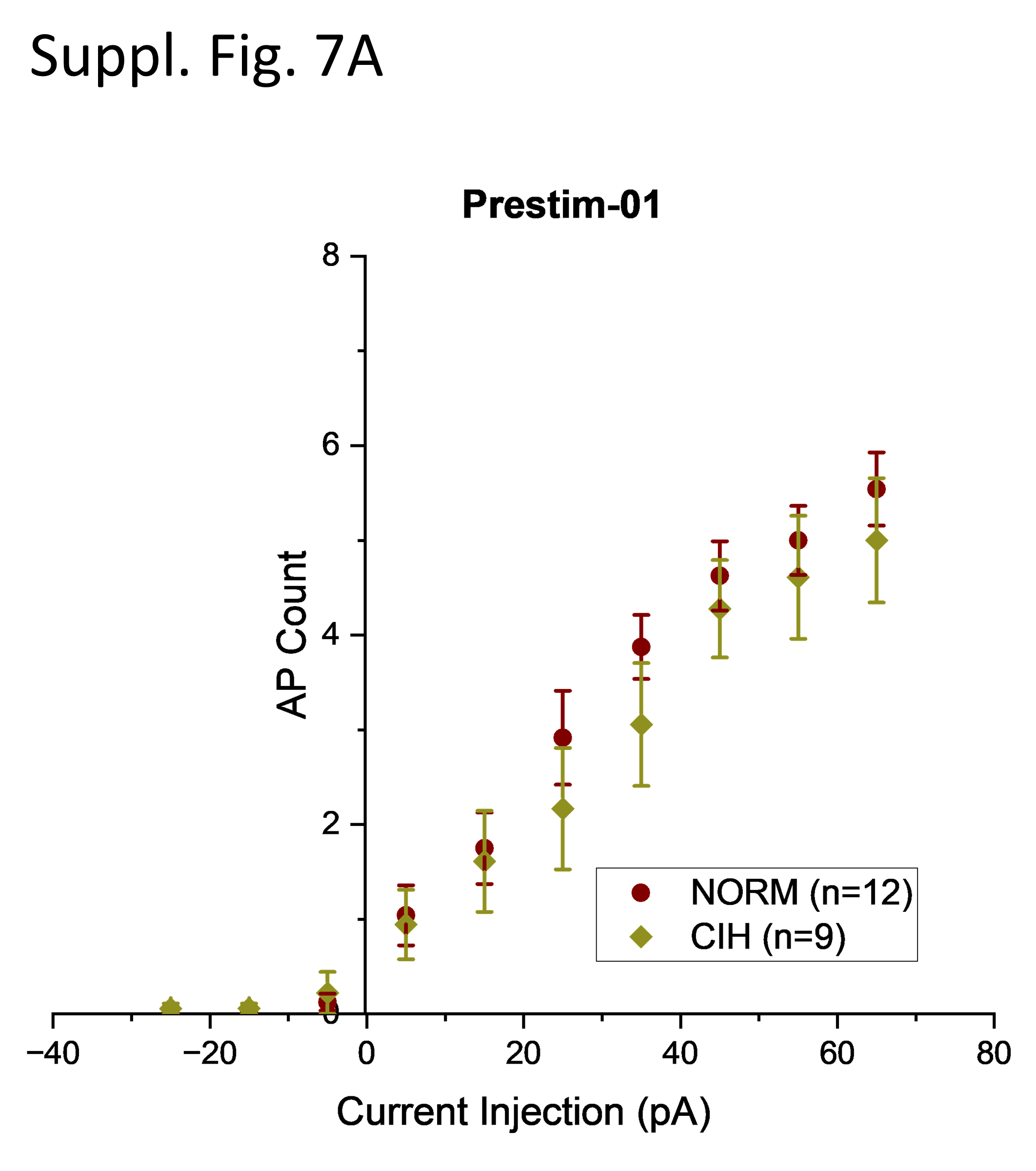

Supplement: Supplementary file 7 — Figure S7. Neuronal excitability before repeated stimulations in normoxic and CIH‐exposed PVN MNCs. The relationship between injected depolarizing current and action potential frequency in normoxic MNCs (maroon circles) and CIH neurons (golden rhombi). Graded current injections evoked graded increases in AP frequency in CIH‐exposed MNCs similarly between NORM and CIH (F(9,69) = 1.36, p = .225) Two‐way repeated measures ANOVA. NORM, normoxic, n = 21 cells, 14 animals. Data are presented as means ± SE. [file JNE-38-e70100-s006.tif]
